# Light-Induced Modulation of Chiral Functions in G-Quadruplex–Photochrome Systems

**DOI:** 10.1021/acs.jpclett.1c02207

**Published:** 2021-09-23

**Authors:** Marta Dudek, Marco Deiana, Kinga Szkaradek, Mikołaj J. Janicki, Ziemowit Pokładek, Robert W. Góra, Katarzyna Matczyszyn

**Affiliations:** † Advanced Materials Engineering and Modelling Group, Faculty of Chemistry, Wroclaw University of Science and Technology, Wyb. Wyspianskiego 27, 50-370 Wroclaw, Poland; ‡ Department of Medical Biochemistry and Biophysics, Umeå University, 90187 Umeå, Sweden; § Theoretical Photochemistry and Photophysics Group, Faculty of Chemistry, Wroclaw University of Science and Technology, Wyb. Wyspianskiego 27, 50-370 Wroclaw, Poland

## Abstract

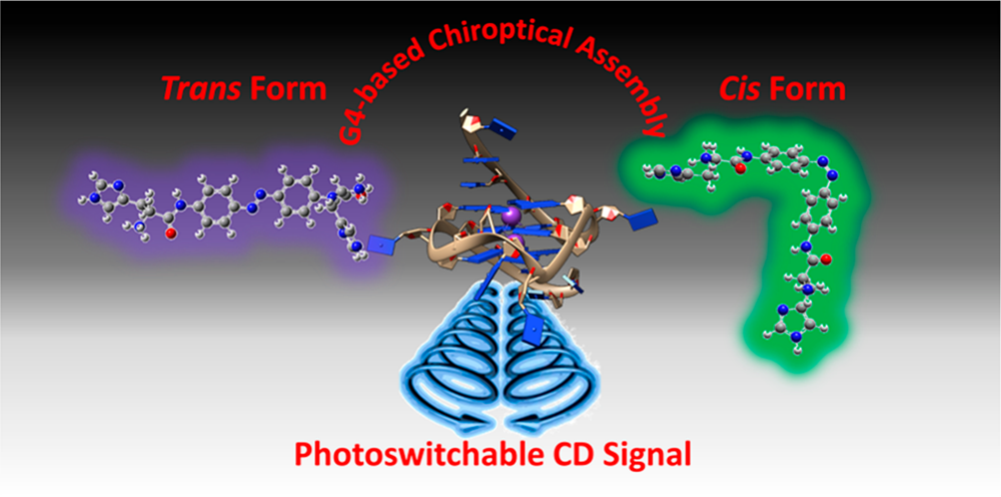

The design of artificially engineered chiral structures has received much attention, but the implementation of dynamic functions to modulate the chiroptical response of the systems is less explored. Here, we present a light-responsive G-quadruplex (G4)-based assembly in which chirality enrichment is induced, tuned, and fueled by molecular switches. In particular, the mirror-image dependence on photoactivated azo molecules, undergoing *trans*-to-*cis* isomerization, shows chiral recognition effects on the inherent flexibility and conformational diversity of DNA G4s having distinct handedness (right- and left-handed). Through a detailed experimental and computational analysis, we bring compelling evidence on the binding mode of the photochromes on G4s, and we rationalize the origin of the chirality effect that is associated with the complexation event.

Photon-fueled therapeutic and imaging modalities rely on the design and construction of tailor-made functional materials capable of executing precisely controlled Ångström-scale structural modifications upon activation with a photonic stimulus.^1,2^ Light offers unparalleled opportunities as a bio-orthogonal spatiotemporally controllable noninvasive regulatory tool for biological applications.^3,4^ While many classes of photoresponsive compounds have been developed,^5^ azobenzene (AB) switches have attracted the most attention in the field of (supra-)molecular recognition.^6−8^ The light-induced *trans*-to-*cis* isomerization of ABs can be achieved by using light of different wavelengths and is accompanied by the simultaneous change in geometry and polarity of the two different isomeric forms.^9,10^ Molecular strategies that target avoiding the use of short-wavelength UV light required to induce isomerization of the azo bond have led to the synthesis of red-shifted ABs that can be switched with the use of the visible light, therefore offering possibilities to control biologically relevant targets in a noninvasive way.^11−13^ Naturally occurring systems such as DNA, RNA, and proteins have been widely implemented as structural platforms to build stimuli-responsive nanostructured hybrid materials with adaptive and reversible functions.^1,8,14,15^ In particular, guanine (G)-rich nucleic acid sequences forming G-quadruplex (G4) motifs are promising programmable metal-mediated assemblies that have been used to design a number of nanometer-scale systems for molecular computing, transport, motor, and biosensing applications.^16,17^ G4s are characterized by stacks of Hoogsteen-bonded guanine tetrads stabilized by centrally octa-coordinated potassium ions.^18^ They also exhibit inherent flexibility and conformation diversity as exemplified by the intramolecular G4 telomeric repeat sequence d[AG_3_(T_2_AG_3_)_3_] which can adopt a variety of strand orientations with parallel, hybrid, and antiparallel architectures.^19,20^ Computational and deep-sequencing studies have unraveled the presence of over 700.000 regions that could potentially form G4s in the human genome.^21,22^ G4 sites are not randomly distributed throughout the genome and are mainly present in the promoters, telomers, and transcription factor binding sites.^23^ The existence of G4-selective binding/stabilizing/unwinding proteins further support the intracellular physiological relevance of G4 motifs.^24,25^ It has been shown that mutations and/or deletions of these proteins lead to a change in G4 formation affecting biological transcriptional pathways or increase genome instability. A set of different experiments by using a G4 specific antibody (BG4), fluorescent reporters, and G4 ChIP-seq showed an increased level of G4 structures in cancer cells compared to normal cells.^22,26−28^ Furthermore, these globular non-B DNA structures have been also shown to play pivotal roles in regulating biological processes such as replication, transcription, translation, and splicing.^18,29^ Therefore, they are recognized as potential therapeutic targets for small-molecule drugs in a range of disease classes including cancer.^18^ Although a plethora of molecules based on different recognition processes^30−36^ have been reported to target G4 motifs, the development of light-responsive G4 ligands has received scant attention to date.^36,37^ Molecular photoswitches based on an azobenzene unit,^6,38^ stilbene-based^39^ and dithienylethene-based ligands,^40^ a (photo)caged G4-binder,^41^ and a photochemically generated π-extended dicationic compound^36^ constitute rare examples of such molecules. Moreover, the design and implementation of molecules with tunable structural chirality to control G4 binding/function is still in its infancy.^6,42−45^

In light of this, herein, we report on the design, synthesis, and characterization of the G4-interactive binding properties of four novel bioinspired AB derivatives with differences in both the modification of the azobenzene ring and substitution pattern of the chiral pendant ligand’s arms (Figure 1, see SI pp S4–S13 for details concerning their synthesis). This rational design strategy affords high structural tunability both in terms of photoswitching properties (almost 80% of *cis* isomer, when **Azo4F-DD/LL-his** is irradiated with light >485 nm, Figure 1C), through the introduction into the azo core of *ortho*(*o*)-fluorine atoms, and chiral functions, through the insertion of *para* (*p*) histidine (**his**) units in both D and L configurations (Figure 1). Moreover, the implementation of positively charged **his** moieties ensures water solubility to the molecules under relevant physiological pH conditions and provides structural recognition motifs via electrostatic attractions with the negatively charged G4 backbone. We show that these chiral photochromes enable control of G4 melting either through the use of light or via the mutual cooperation between the inherent G4 chirality and spatial configuration of the pendant ligand’s arms. Furthermore, the noncovalent molecular recognition mediated by the chiral switches to the inherent chiral G4 matrices led us to discover a close connection between their sequence-specific structural chirality and the amplification of the resulting circular dichroism (CD) signal. The resulting hybrid photochrome–G4 chiral structures can be reversibly modulated through photoexcitation, and with the help of quantum-chemical calculations, we explored the origins of that phenomenon.

**Figure 1:**
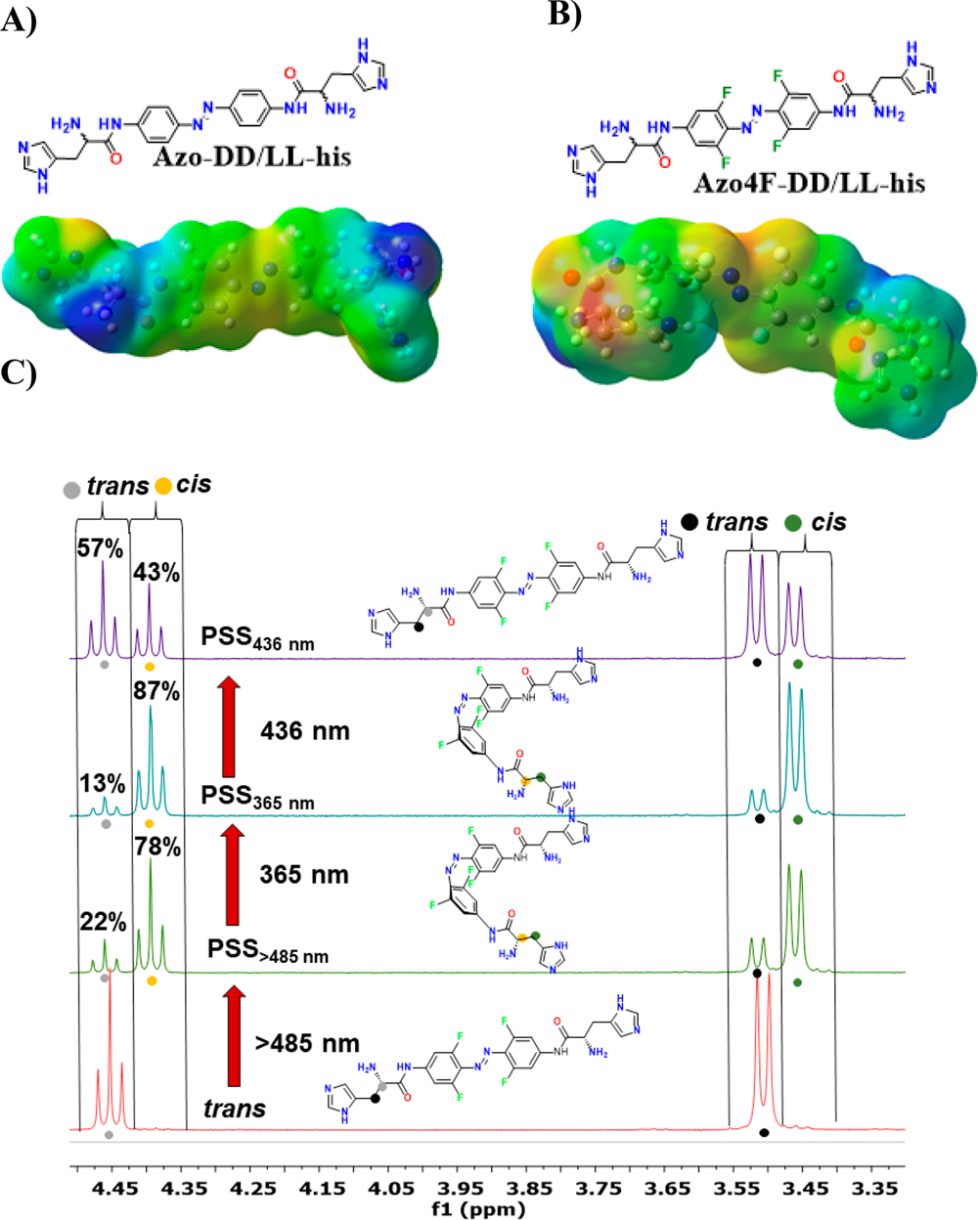
(A, B) Synthesized and investigated switches in *trans* form along with their optimized structures and corresponding electrostatic potential maps. (C) Quantification of the PSSs of **Azo4F-LL-his** (*c*_**Azo4F-LL-his**_ = 3 mM, in water at 25 °C) by ^1^H NMR spectroscopy. The contents of *trans* and *cis* forms in solution were calculated from the intensity ratios of the integrals of the corresponding peaks.

The electronic absorption spectrum of **Azo-DD/LL-his** in *trans* form features a strong π → π* absorption band at short wavelengths (λ_π→π*_ ≈ 359 nm) and a weaker and red-shifted n → π* tail (Figure 2A,C). The UV/vis spectrum of **Azo4F-DD/LL-his** resembles that of the parent ABs (**Azo-DD/LL-his**) with a π → π* transition centered at 352 nm but with a defined n → π* band at 441 nm (Figure 2B,D). Irradiation of **Azo-DD/LL-his** with UV light at 365 nm causes *trans* → *cis* isomerization, resulting in the simultaneous decrease and blue shift of the π → π* band (λ_π→π*_ ≈ 321 nm) along with the formation of the n → π* band (λ_n→π*_ ≈ 436 nm). This evolution is typically observed in ABs and generates a photostationary state (PSS) containing at least 77% of the corresponding *cis*-isomer as derived from HPLC traces (Figure S13). Irradiation of **Azo4F-DD/LL-his** with a 365 nm UV light provides qualitatively similar results as observed for **Azo-DD/LL-his** and generates a PSS containing 87% of the corresponding *cis*-isomer as calculated from ^1^H NMR (Figure 1C). However, the UV-mediated blue-shift of the n → π* band in the fluorinated compounds (Δλ_n→π*_ = 12 nm) ensures an effective separation of the n → π* bands of the two isomers (Figure 2D). This feature enables the switching of the isomeric forms of **Azo4F-DD/LL-his** with visible light in both directions, producing PSSs containing 78% of the *cis* form with λ_exc_ > 485 nm and 43% of *cis*-isomer with blue light (436 nm) (Figure 1C). Remarkably, the *cis* form of **Azo4F-DD/LL-his** is thermally and energetically more stable at 25 °C (with half-life τ_1/2_ ca. 47 h and activation energy *E*_a_ ∼ 99.0 kJ/mol) in comparison to **Azo-DD/LL-his** (τ_1/2_ ca. 0.1 h and *E*_a_ ∼ 83.0 kJ/mol) (see SI pp S15–S17).^12,46^

**Figure 2:**
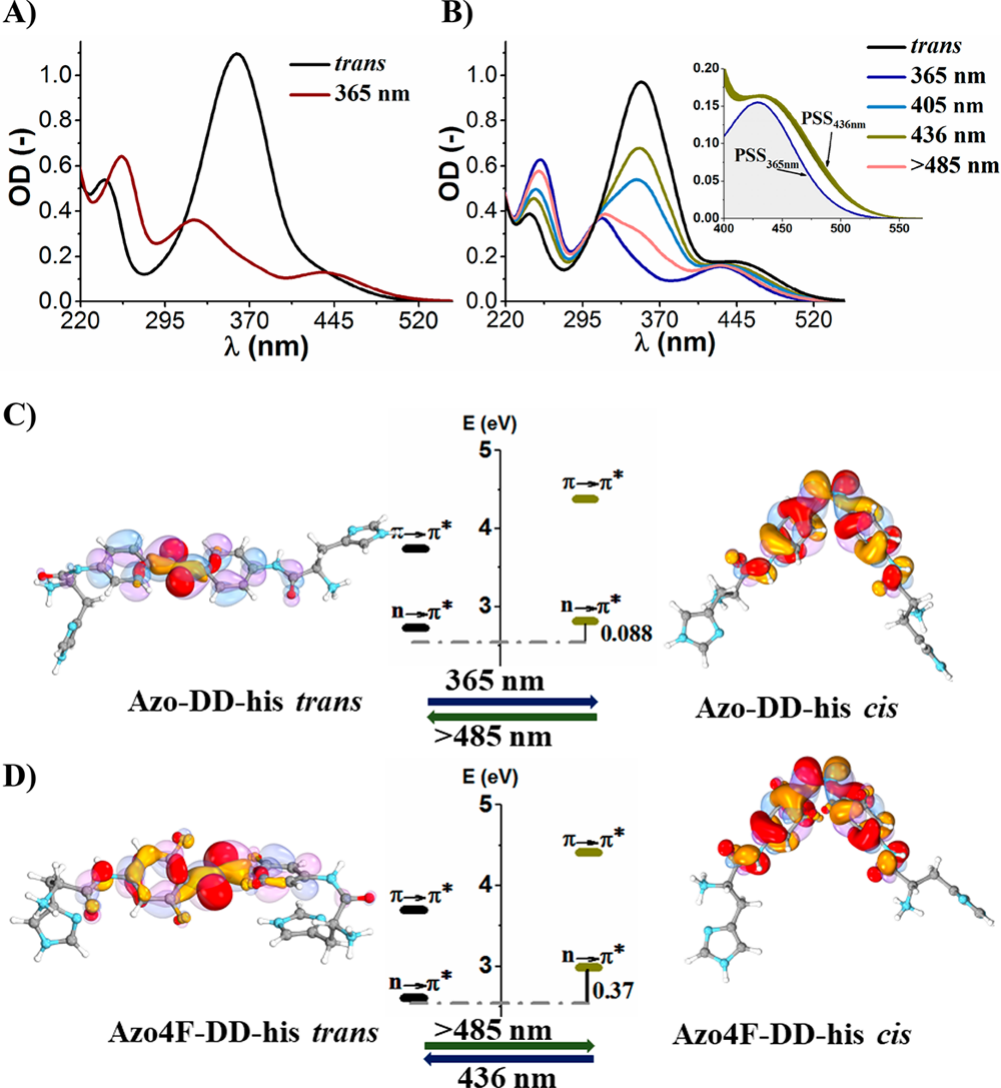
Absorption spectra of (A) **Azo-DD-his** and (B) **Azo4F-DD-his** recorded in water solutions under excitation with different wavelengths of light (*c*_**Azo-DD-his**_ and *c*_**Azo4F-DD-his**_ = 30 μM). Inset in (B) shows the n → π* band of **Azo4F-DD-his** in the spectral region between 400 and 550 nm. Panels (C) and (D) show the computed energetic diagrams in the Franck–Condon region of the two lowest-lying excited states having π → π* and n → π* character. The corresponding n and π* orbitals for the latter are shown in yellow-red and purple-blue, respectively. The results of calculations were obtained at the ωB97xD/def2-TZVP level (see SI pp S33 for details).

The interesting structural and optical properties of the designed ABs prompted us to study their recognition toward biologically relevant DNA G4s which adopt diverse conformations and strand orientations. In particular, right-handed antiparallel (Bom17), hybrid (Tel-22), and parallel (*c-MYC* Pu22) G4-forming sequences as well as a left-handed parallel G4 (ZG4) and a double-stranded B-DNA (dsDNA) were used as biological templates in stability measurements with the switches. The proper folding of all the used DNA sequences was probed by CD measurements (Figure S17).

The binding performances of the switches to duplex and G4 templates were investigated by CD- (or UV-) based thermal melt (*T*_m_) assays, in which molar ellipticity or absorption were measured as a function of increasing temperature (see SI pp S19–S22). In particular, the melting temperature changes (Δ*T*_m_) of Bom17 in the presence of **Azo4F-DD-his** (*trans*) or **Azo4F-LL-his** (*trans*) increased by ∼9 and 6 °C, respectively, showing a fair chiral selectivity between the two enantiomers (Figure 3A). A similar trend was obtained when using the parent compounds **Azo-DD/LL-his** (*trans*), even if the extent of thermal stabilization on the Bom17 template was reduced to ∼5 and 4 °C for **Azo-DD-his** and **Azo-LL-his**, respectively. On the other hand, the same experiment performed with **Azo4F-DD/LL-his** or **Azo-DD/LL-his** (*trans*), in the presence of left-handed parallel ZG4, showed limited or no structural stabilization (Figure 3B). This result suggests that changes in the handedness of the G4 chirality affect the recognition abilities of the chiral switches. Conversely, the melting temperature of the hybrid (Tel-22) and parallel (*c-MYC* Pu22) G4s in the presence of these molecules in *trans* form increased by ∼8–9 °C or ∼9–12 °C, respectively, but no chiral recognition was observed, with the induced G4 thermal stabilization being almost identical for both enantiomers (Figure S24). Finally, little to no stabilizing properties were observed for the photochromes in *trans* form in the presence of duplex DNA whether in L or D configuration (Figure S24).

**Figure 3:**
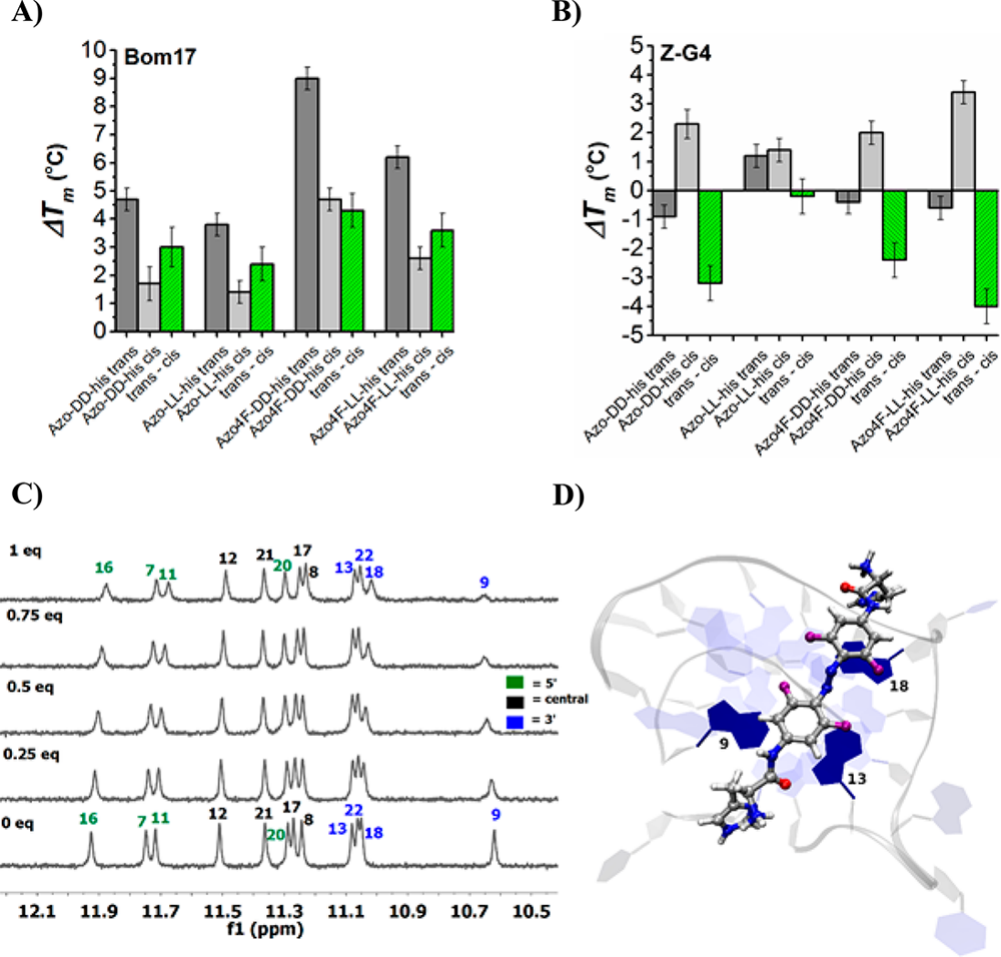
(A, B) Induced thermal stabilization by **Azo-DD/LL-his** and **Azo4F-DD/LL-his** (*trans* and *cis*-rich PSS) on right-handed Bom17 or left-handed Z-G4. Results are presented as an average of three independent experiments. The *trans*–*cis* underlines the thermal stabilization difference between the *trans* isomer and the *cis*-rich PSS. The concentration of KCl in Tris-HCl buffer (10 mM) was 5 mM for Z-G4 and 15 mM for Bom17. (C) ^1^H NMR spectra of the G-tetrad imino protons in the absence (0.0 equiv) and presence (0.25, 0.5, 0.75, and 1.0 equiv) of **Azo4F-DD-his***trans*. (D) Representative binding site of **Azo4F-DD-his***trans* during docking with the 3′-end of c-MYC Pu22 (cf. SI pp S37 for details).

Unfortunately, the low thermal stability of the *cis* isomer and the associated fast *cis–trans* conversion of **Azo-DD/LL-his** in the temperature range in which the dsDNA and *c-MYC* Pu22 melts limits possibility of investigation of the stabilizing ability of the *cis* isomer toward these DNA structures. On the other hand, **Azo4F-DD/LL-his**, as *cis*-rich PSS, showed high thermal stability and could, therefore, be used in all photodependent thermal melt assays. Indeed, the *in situ*, induced *trans*-to-*cis* isomerization of **Azo4F-DD/LL-his** with visible light > 485 nm stabilized most of the right-handed G4 templates regardless of the folded topology but only to such an extent that the melting temperature was lower compared to the related *trans* form. It is worth noting that, in the presence of left-handed Z-G4, the *cis*-rich PSS induced a higher degree of thermal stabilization in comparison to the *trans* form. Overall, these data support the possibility to tune the interactive stabilizing properties of the switches through the mutual cooperation between chiral functions and light-induced mechanical forces.

To explore the structural origin of the interaction between **Azo4F-DD-his** (*trans* and *cis*-rich PSS) and G4s, we performed ^1^H NMR titration experiments by using the model system *c-MYC* Pu22 (Figures 3C, S25, and 26). Free *c-MYC* Pu22 forms a single G4 conformation as shown by the well-resolved imino proton peaks associated with the three G-tetrad layers.^47^ Addition of an equimolar concentration (1.0 equiv) of **Azo4F-DD-his***trans* to *c-MYC* Pu22 resulted in a marked decrease in intensity of the imino protons assigned as G9 and G16 along with a chemical shift perturbation of G20 and G18 indicating binding of **Azo4F-DD-his***trans* at both the 5′ and 3′ G-tetrad ends (Figure 3C). Interestingly, titration experiments performed with **Azo4F-DD-his** as *cis*-rich PSS resulted in minimal chemical shift perturbations of the imino protons associated with the 5′-end. Conversely, the imino protons (G9, G18, G22, and G13) associated with the 3′-end were all largely affected by the presence of the ligand as well as G17 that is located within the central G-tetrad layer. Since G17 is located above G18, we speculate that the azobenzene ligand’s arms can be sandwiched between these two G4-tetrads affecting both the guanines. Next, we performed *in situ* photoisomerization of **Azo4F-DD-his** bound to *c-MYC* Pu22 (Figure S26). As expected, the light-induced *trans*-to-*cis* conversion of **Azo4F-DD-his** led to similar chemical shift perturbations as observed for **Azo4F-DD-his***cis*-rich PSS providing solid evidence for the possibility to tune the interactive binding process/localization of the azobenzene in a light-controlled manner. From these data, it appears that the more hydrophobic and planar *trans* isomer can stack onto the G-tetrad ends with great efficiency. On the other hand, the bent and more hydrophilic *cis* isomer is partially displaced by these terminal G4-sites providing a more heterogeneous binding event that may involve further interactions with the grooves/loops or flanking residue of the G4 structure. The experimental observations on the ability of the azobenzene to stack onto the 5′- and 3′-ends of *c-MYC* Pu22 were also mechanistically investigated by theoretical methods (Figure 3D, SI pp S37). Calculations showed coordination of the ABs at the G4-tetrad ends in fair agreement with the experimental results.

The possibility to control the structural and chiral properties of the switches with the ability to tailor the topological DNA assemblies prompted us to investigate the opportunity to create a hybrid chiroptical system tunable with light. We focused our attention on **Azo-DD/LL-his**, which exhibited a nearly symmetric but inverted CD profile whether in *trans* form or *cis*-rich PSS (Figure S18). Addition of G4s to **Azo-DD/LL-his** resulted in an amplified CD band that spanned the spectral region (∼300–500 nm) where the G4s are CD silent and only the switches absorb light (Figures 4A and S27–S30). Monotonic changes in the CD bands of the switches were observed on further increase of the G4 concentration until a plateau was reached. No chiral spectral changes in the **Azo-DD/LL-his**-dsDNA (Figures 4B and S31) systems were observed, supporting the selectivity of these molecules against G4 structures. Since addition of up to 3 equiv of **Azo-DD/LL-his** to G4s did not induce structural modification or topological changes in the biological templates (Figures S32–S41), it is apparent that the observed CD signal resulted from a specific structural orientation imposed by the G4 structures on the switches. Therefore, we carried out quantum-chemical calculations to ascertain the molecular origin of the characteristic CD bands (Figure 4C–E and SI pp S33). Using metadynamics simulations, we explored a model system containing the 3′-end part of *c-MYC* Pu22 and **Azo-DD-his** (*trans*) (Figure 4C). This approach allowed us to find a set of plausible conformers of **Azo-DD-his***trans* that can occur in the host–guest complex (Figure 4D). The most representative structure of **Azo-DD-his***trans* from the complex was used to simulate the CD spectra using the TD-DFT method (Figure 4E). Since **Azo-DD-his***trans* is CD active, we calculated its circular dichroism spectral signatures (Figure 4E), which exhibit a negative dichroic band having a maximum at 330 nm, which is in a good agreement with the experimental results (Figure S18). Coordination of the azo molecule with the G4 template can lead to structural perturbation and/or rearrangement on the substituents on the AB scaffold along with the formation of specific geometrical conformations that can alter the resulting CD spectral profile of the system. Indeed, the experimental CD features showing both the sign change in the Cotton effect of the band centered at ∼360 nm and the appearance of a new and well-structured negative band at ∼450 nm for the **Azo-DD-his***trans*-*c-MYC* Pu22 system were systematically reproduced by theoretical calculations (Figure 4A,C–E). In particular, complexation of **Azo-DD-his** with the 3′-end of *c-MYC* Pu22 induced the formation of a stacked arrangement of the histidine moiety with the phenyl ring of the azobenzene scaffold, which translates into a high dissymmetry supra-structure showing an amplified negative CD band centered at ∼433 nm (Figure 4C–E), corresponding to the experimental feature centered at ∼450 nm (Figure 4A).

**Figure 4:**
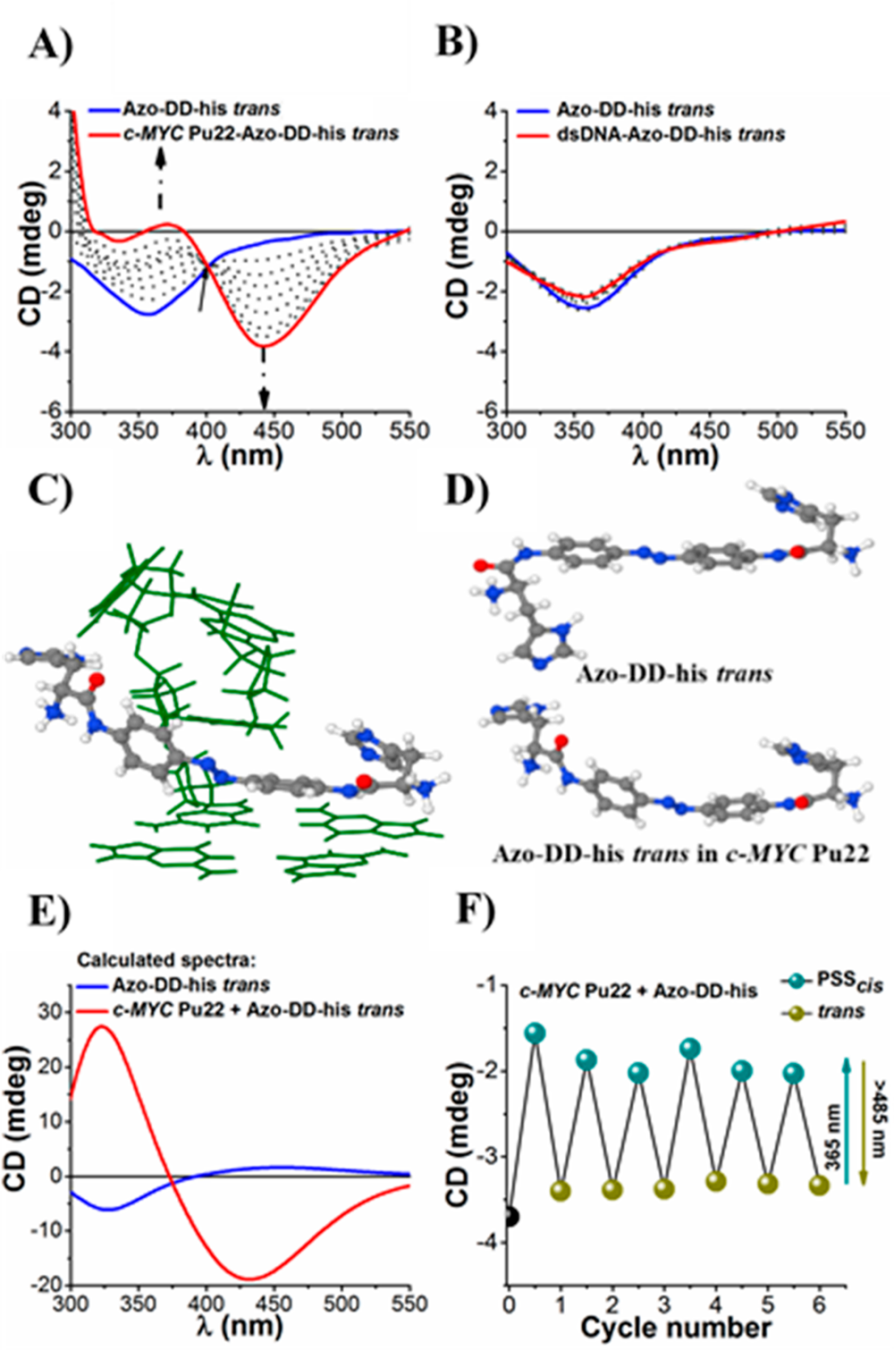
Experimental ECD spectra resulting from the complexation of **Azo-DD-his***trans* with (A) *c-MYC* Pu22 and (B) dsDNA. *c*_**Azo-DD-his***trans*_ = 50 μM and *c*_*c-MYC*Pu22/dsDNA_ = from 0 to 20 μM at 20 °C. The arrows aim to show the evolution of the binding profile and the appearance of isodichroic points. (C) The ground-state structure of the complex between the 3′-end of *c-MYC* Pu22 and **Azo-DD-his***trans* obtained from metadynamics simulations as well as (D) its comparison with the ground-state structure of the free molecule. (E) Calculated circular dichroism spectra of **Azo-DD-his***trans* in its free and bound state with *c-MYC* Pu22 using the ωB97xD/def2-TZVP/PCM method. (F) ECD photoswitching monitored at 450 nm of the *c-MYC* Pu22-**Azo-DD-his** complex (at 20 °C) by alternating cycles of UV (365 nm) and visible light (>485 nm).

Interestingly, the magnitude but not the shape of the signal at 450 nm could be tuned by changing the state of the switch from *trans* to *cis*-rich PSS (Figure 4F). In particular, irradiation with UV light resulted in a reduction of the CD signal, whereas the use of visible light amplified the intensity of the CD band. This mechanism provided the possibility to reversibly tune the chiroptical response of the G4–photochrome systems by light. Several reversible switching cycles with a limited photochemical fatigue could be achieved. It turns out that the amplified CD signal can be controlled by simply changing the energy of photons after irradiation for a selected period of time.

In summary, we have developed selective G4-targeted photoactivated azobenzene molecules whose binding interactions can be modulated through the insertion of chiral functions and light irradiation. The mutual cooperation between the mirror image dependence of right- and left-handed G4s and the state/chirality of the switches provides findings for the application of azo molecules as chiral selective G4-binders. Moreover, the assembly resulting from the binding of photochromes to G4s enables one to build up a chiroptical system tunable through the application of light having different wavelengths. Computational mechanistic analysis sheds light on the molecular rearrangement of the switch complexed with G4 and the origin of the associated amplified chiral signal. These findings open up new directions for adaptive, bioinspired nano systems and biological regulations involving G4 structures.

## Supporting Information Available

Experimental procedure, synthesis, and photoisomerization studies as well as thermal stability of the synthesized azobenzene derivatives, DNA characterization, ECD spectra of **Azo-LL/DD-his** (*trans*/*PSS*_*cis*_) and **Azo4F-LL/DD-his** (*trans*/*PSS*_*cis*_), melting studies, NMR titration experiments, ECD spectra of **Azo-LL/DD-his** complexed with duplex and quadruplexes, duplex and quadruplex conformational changes, and computational details and results (PDF)
