# Supplementary material for: Light-Induced Modulation of Chiral Functions in G-Quadruplex–Photochrome Systems
Source: J Phys Chem Lett. 2021 Sep 23;12(39):9436–41. doi: 10.1021/acs.jpclett.1c02207 (PMC8503878; doi:10.1021/acs.jpclett.1c02207)
Supplement: Supplementary file 1 — jz1c02207_si_001.pdf [file jz1c02207_si_001.pdf]

# *Supporting Information for*

# Light-Induced Modulation of Chiral Functions in G-Quadruplex-Photochrome Systems

*Marta Dudek,[a*] Marco Deiana,[b] Kinga Szkaradek,[c] Mikołaj J. Janicki,[c] Ziemowit Pokładek,[a] Robert W. Góra[c] and Katarzyna Matczyszyn[a*]*

[a] Advanced Materials Engineering and Modelling Group, Faculty of Chemistry, Wroclaw University of Science and Technology, Wyb. Wyspianskiego 27, 50-370 Wroclaw, Poland

[b] Department of Medical Biochemistry and Biophysics, Umeå University, 90187 Umeå, Sweden

[c] Theoretical Photochemistry and Photophysics Group, Faculty of Chemistry, Wroclaw University of Science and Technology, Wyb. Wyspianskiego 27, 50-370 Wroclaw, Poland

Corresponding Authors

*E-mail: katarzyna.matczyszyn@pwr.edu.pl and marta.ziemianek-dudek@pwr.edu.pl

**Table of Contents:**


1. Materials and methods S2
2. Synthesis and characterization S4
3. Photoisomerization studies S14
4. Thermal stability S15
5. G4 studies S17
   5.1 G4 characterization S17
   5.2 ECD spectra of Azo-LL/DD-his and Azo4F-LL/DD-his S18
   5.3 Melting studies S19
   5.4 NMR studies S23
   5.5 ECD spectra of Azo-LL/DD-his complexed with duplex and quadruplexes S25
   5.6 Duplex and quadruplexes conformational changes S28
6. Theoretical calculations S33
   6.1 Conformational sampling S33
   6.2 Molecular Docking S37
7. References S38


# MATERIALS AND METHODS

**Solvents and reagents** were purchased from commercial suppliers and were used as supplied. Column chromatography was performed using silica gel (Acros 60, 40–60 mesh). Unless otherwise noted, all reactions were carried out under normal conditions. Oligonucleotides were purchased from Sigma Aldrich, diluted with Milli-Q water to a concentration of 2 mM and stored in the fridge. A stock solution of 1 M KCl in water was prepared using solid KCl. A solution of Tris (1M) was adjusted with HCl to desired pH = 7.4.

**NMR-spectra** were recorded on a Bruker AvanceTM 600 MHz spectrometer or on a JEOL 400 MHz spectrometer at 25 °C using residual protonated solvent signals as internal standards for $^{1}$H- and $^{13}$C-spectra.

**High resolution mass spectra** (HRMS) were conducted with a WATERS LCT Premier XE mass spectrometer (ESI).

**High Pressure Liquid Chromatography** (HPLC) was done using a Knauer Azura ASM 2.1L equipped with a C18 column (Thermo Scientific, Hypersil Gold 12 μm, 250 mm × 20 mm) in water/acetonitrile (0.05% TFA).

**Absorption spectra**. UV–Vis experiments were carried out on a PerkinElmer Lambda 20 UV–Vis spectrometer connected with a cooling system or on a Hitachi U-2900 spectrophotometer at 25 °C. All optical measurements were performed in quartz cell cuvettes with conventional path lengths of 10 mm.

**Composition of the photostationary states (PSSs)**. The photoinduced isomerization reactions of **Azo-DD/LL-his** and **Azo4F-DD-his** were performed by using a UV Spot Light Source (Hamamatsu Photonics K.K., model: L9588-04) equipped with filters operating at: 365 nm, 436 nm and >485 nm. The composition of the photostationary states ($PSS_{cis}$ and $PSS_{trans}$) were determined either by HPLC or $^1$H NMR spectroscopy for **Azo-DD/LL-his** and for **Azo4F-DD/LL-his**, respectively. The 30 μM water solution of **Azo-DD-his** was irradiated with UV light (365 nm for 10 minutes) and then immediately injected on the HPLC column (cooled to 10 °C to prevent *cis-trans* thermal relaxation). The experiment was performed using a gradient from water to water/MeCN (0.5/0.5) in 5 minutes (flow: 0.5 mL/min, columm: C18). The PSS composition was determined by integration of the UV signal taken at the isosbestic point. The PSSs of **Azo4F-LL-his** (3 mM in water solution at 25 °C) were measured by NMR spectroscopy by irradiating the sample, each time for 15 minutes, with different excitation sources: >485 nm, 365 nm and 436 nm, prior recording the NMR spectra. The composition of the PSS was calculated from the intensity ratios of the integrals of the corresponding peaks.

**Thermal stability**. To analyze the thermal relaxation process from the *cis*-rich PSS to the *trans* form, the absorbance changes were measured at different temperatures. First, both **Azo-LL/DD-his** and **Azo4F-LL/DD-his** (30 μM in $H_2O$) were irradiated with UV light (365 nm) for 15 min to convert the *trans* form into the *cis* form. Then, absorbance readings were taken at the absorption maximum with 5 s intervals at 15 °C, 20 °C, 25 °C and 30°C for **Azo-LL/DD-his** and at 50 °C 60 °C, 70 °C and 75 °C for **Azo4F-LL/DD-his**.

**ECD spectra** were measured with a Jasco J-1500 spectropolarimeter (JascoInc, USA) equipped with the JascoPeltier-type temperature controller (CDF-426S/15) at 20 °C and are presented as a sum of 2 (for the *cis*-rich PSS to minimize the effect of *cis-trans* isomerization) or 3 accumulations. Before use, the optical chamber of the CD spectrometer was deoxygenated with dry nitrogen and was held under nitrogen atmosphere during the measurements. Appropriate references were subtracted from the obtained CD spectra.

**Melting studies.** Melting experiments were conducted with a Jasco J-1500 spectropolarimeter (JascoInc, USA) equipped with the JascoPeltier-type temperature controller (CDF-426S/15) by recording both the UV/Vis and CD spectra. CD melting assays were performed at a fixed G4 concentration (2 μM), either without or with a fixed concentration (8 μM) of the photochromes. Melting studies were performed in Tris-HCl buffer (10 mM) supplemented with 15 mM KCl (Bom17 systems) or 5 mM KCl (*c-MYC* Pu22, Tel-22, Z-G4 and dsDNA systems). The obtained plots enabled calculation of the $T_m$ values by using sigmoidal nonlinear curve-fitting procedures. Note, that the system: **Azo-DD/LL-his** *cis*/Bom17, Tel-22, Z-G4 was irradiated for 40 s with UV light (365 nm) at each interval of temperature before recording the spectral signature.

**NMR titration.** *c-MYC* Pu22 ($c_{c\text{-}MYC\ \mathrm{Pu22}}$ = 300 μM, tris buffer 10 mM, 33 mM KCl, 10% $D_2O$, pH = 7.4) was titrated with increasing amount of **Azo4F-DD-his** in *trans* form and *cis*-rich PSS.

# SYNTHESIS AND CHARACTERIZATION

## General Information

The AB derivatives (**Azo-DD/LL-his** and **Azo4F-DD/LL-his**) were attempted to synthetize through standard peptide bond synthesis protocols with HATU as a coupling agent (Scheme S1).[1] However, this approach, after removing the protecting group: Fmoc with DEA and Trt with TFA, enabled to get **Azo-LL-his** or **Azo-DD-his** without fluorine atoms at *ortho* position. Such a behavior can be explained based on the lower nucleophilicity of the amine (caused by a strong negative inductive effect of fluorine) of compound **8** compared to compound **1** (Figure S42-43). To rationalize this hypothesis, we performed the Natural Bond Orbital (NBO) analysis for minimum energy structures located at the CPCM(water)/ωB97xD/def2-TZVP level. Indeed, the calculated charge at the nitrogen of the amino group is less negative for compound **8** (-0.758 e) than for compound **1** (-0.776 e). Moreover, the coupling between nitrogen and the aromatic ring, as estimated by the second-order perturbation theory analysis of the corresponding Fock matrices in NBO basis, is more pronounced for azobenzene substituted with fluorine atoms at *ortho* position (67.95 kcal/mol for **8** vs. 60.38 kcal/mol for **1**). Finally, the NBO analysis indicates that the lone-pair orbital energy of the amino nitrogen is higher for compound **8** (-8.98 eV) than for **1** (-8.82 eV). All these factors imply that compound **8** is less reactive than compound **1**. Thus, another strategy was implemented to synthesize compounds **15** and **16**. This approach (Scheme S2) relies on Buchwald type coupling between **6** and **11** or **12**,[2] followed by amidation of carboxylic acid derivatives (**9**, **10**) in the presence of ammonium chloride, using HATU. Then, the protecting groups (Trt and Boc) were cleaved by trifluoroacetic acid to give the final molecules **15** and **16** in 38% and 36% yields, respectively.

**Azo-LL/DD-his** (**4** or **5**) were synthesized according to the route depicted in Scheme S1. The starting compound **1** was synthesized according to the previously published procedure[3] while, **2** and **3** were obtained from commercial supplier.

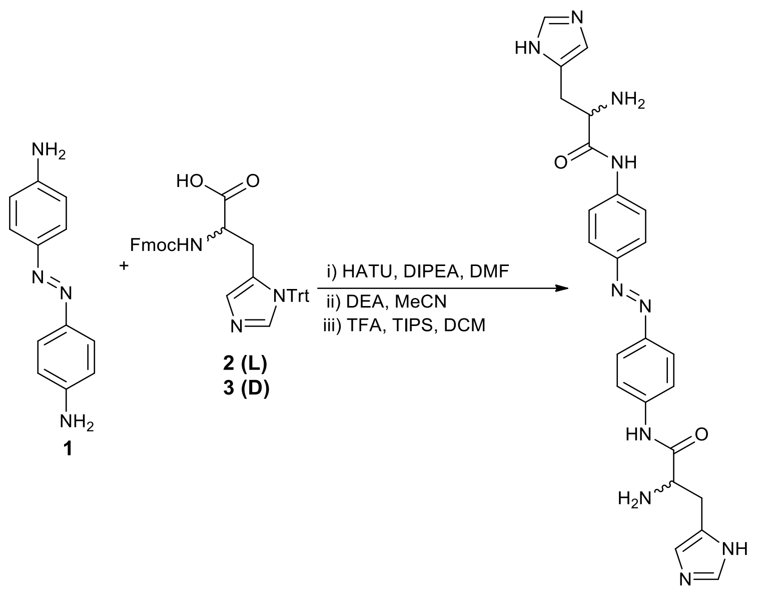


**4 (LL) or Azo-LL-his**
**5 (DD) or Azo-DD-his**

**Scheme S1**. Synthetic route of **Azo-LL-his** (**4**) and **Azo-DD-his** (**5**).

## Synthesis of 4 and 5

Into a solution of Fmoc-His(Trt)-OH (L for **4** and D for **5**) (0.7 g, 1.13 mmol, 2.4 equiv.) in DMF (10 mL), DIPEA (0.29 g, 2.25 mmol, 4.8 equiv.) and HATU (0.43 g, 1.13 mmol, 2.4 equiv.) were added followed by the further addition, after a few seconds, of 4,4'-diaminoazobenzene (0.10 g, 0.47 mmol, 1 equiv.). The reaction mixture was stirred overnight at room temperature. Then the mixture was diluted with water and extracted with ethyl acetate (3 x 50 mL). Organic phases were combined, dried over $MgSO_4$ and the solvent was removed under reduced pressure. Without further purification, F-moc protection group was removed by adding the obtained solid into a solution based on diethylamine (DEA) (0.8 mL) and MeCN (0.8 mL). The mixture was stirred for 2 h and concentrated under reduced pressure. The obtained orange solid was dissolved in the mixture containing: 10 mL DCM, 1 mL of TFA and 0.1 mL of TIPS to remove Trt group. The reaction mixture was stirred for additional 2 hours, then the solvent was removed under vacuum and the crude product was purified by HPLC using a gradient from water to water/MeCN (0.1/0.9) in 25 minutes. **4**, **5** were obtained as orange powders (4: 0.19 g, 36%, 5: 0.2 g, 38%).

Spectral data for **Azo-LL-his** (**4**):

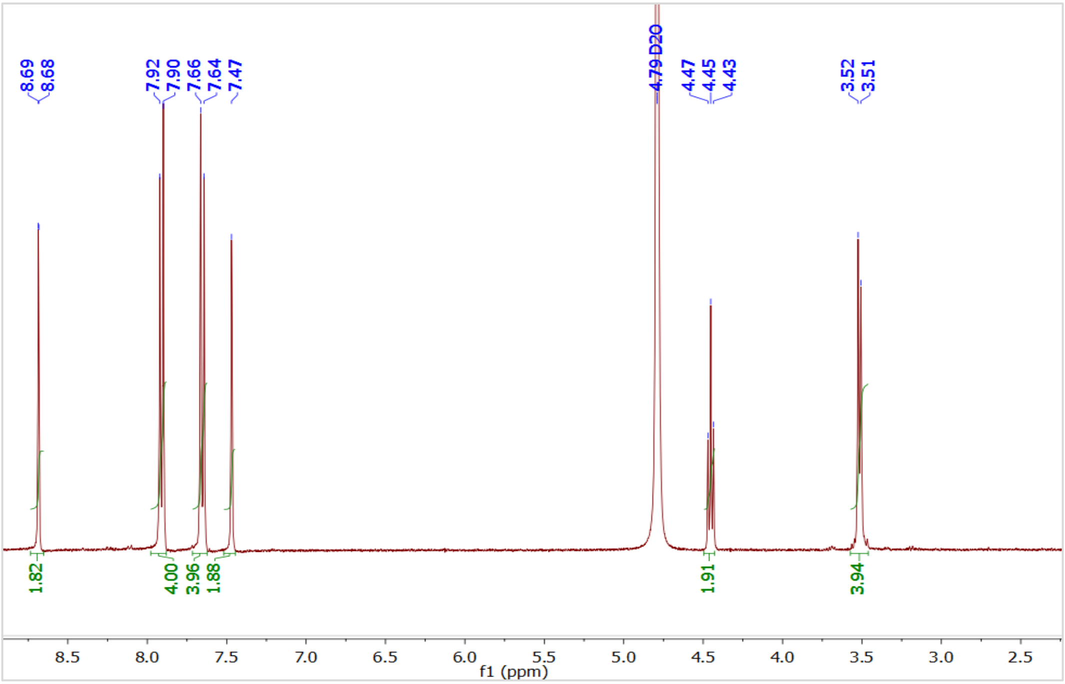


**Figure S1.** $^{1}H$ NMR spectrum of **Azo-LL-his** (**4**).

**$^{1}H$ NMR (400 MHz, Deuterium Oxide) δ:** 8.68 (d, $J$ = 1.2 Hz, 2H), 7.95 – 7.86 (m, 4H), 7.69 – 7.62 (m, 4H), 7.47 (s, 2H), 4.45 (t, $J$ = 7.0 Hz, 2H), 3.52 (d, $J$ = 7.0 Hz, 4H).

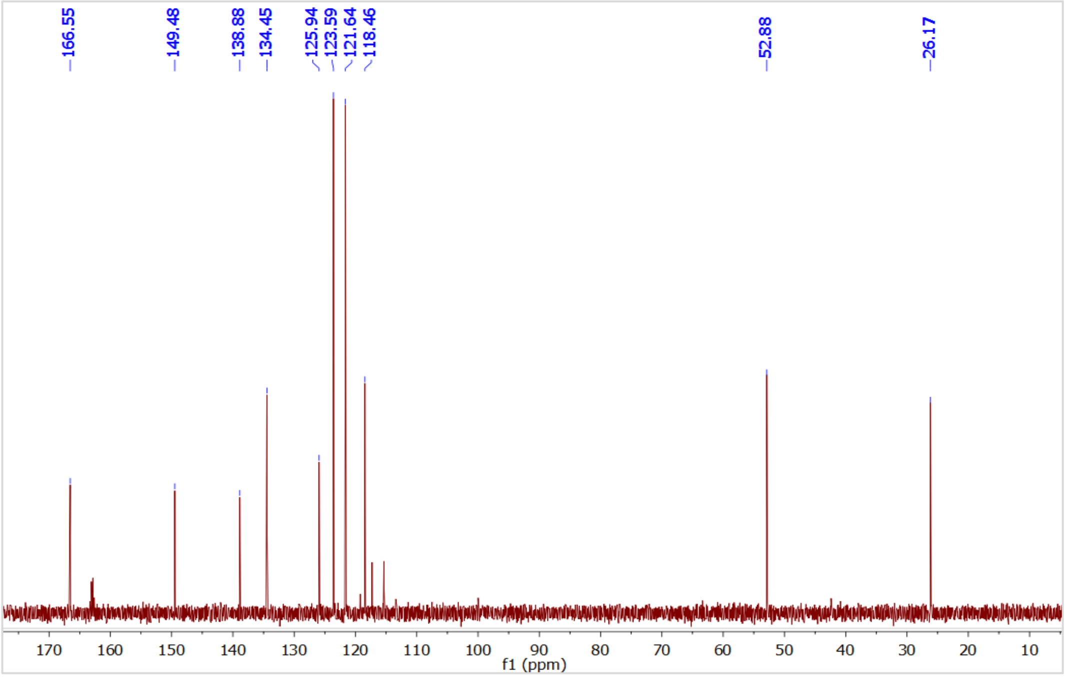


**Figure S2**. $^{13}C$ NMR spectrum of **Azo-LL-his** (**4**).

**$^{13}C$ NMR (151 MHz, Deuterium Oxide) δ:** 166.6, 149.5, 138.9, 134.5, 125.9, 123.6, 121.6, 118.5, 52.9, 26.2.

**HRMS m/z (ESI)**: $C_{24}H_{26}N_{10}O_2$ $[M+H]^+$, calculated: 487.2318, found: 487.2322.

Spectral data for **Azo-DD-his** (**5**):

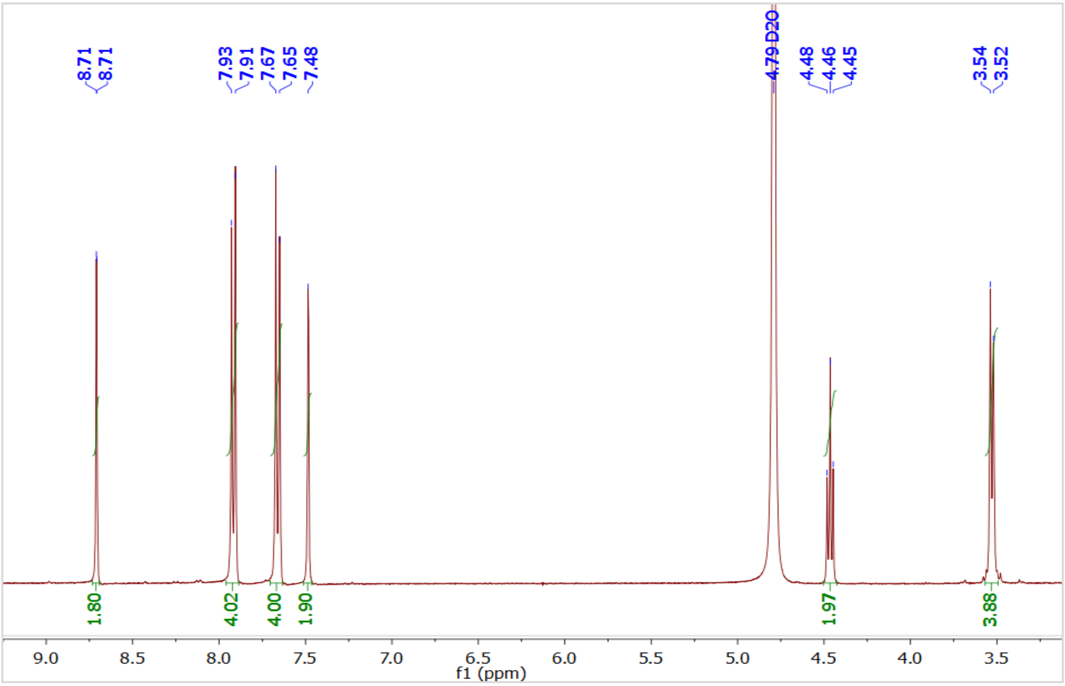


**Figure S3**. $^{1}$H NMR spectrum of **Azo-DD-his** (**5**).

**$^{1}$H NMR (400 MHz, Deuterium Oxide) δ:** 8.71 (d, $J$ = 1.3 Hz, 2H), 7.95 – 7.89 (m, 4H), 7.70 – 7.63 (m, 4H), 7.48 (s, 2H), 4.46 (t $J$ = 7.0 Hz, 2H), 3.53 (d, $J$ = 7.0 Hz, 4H).

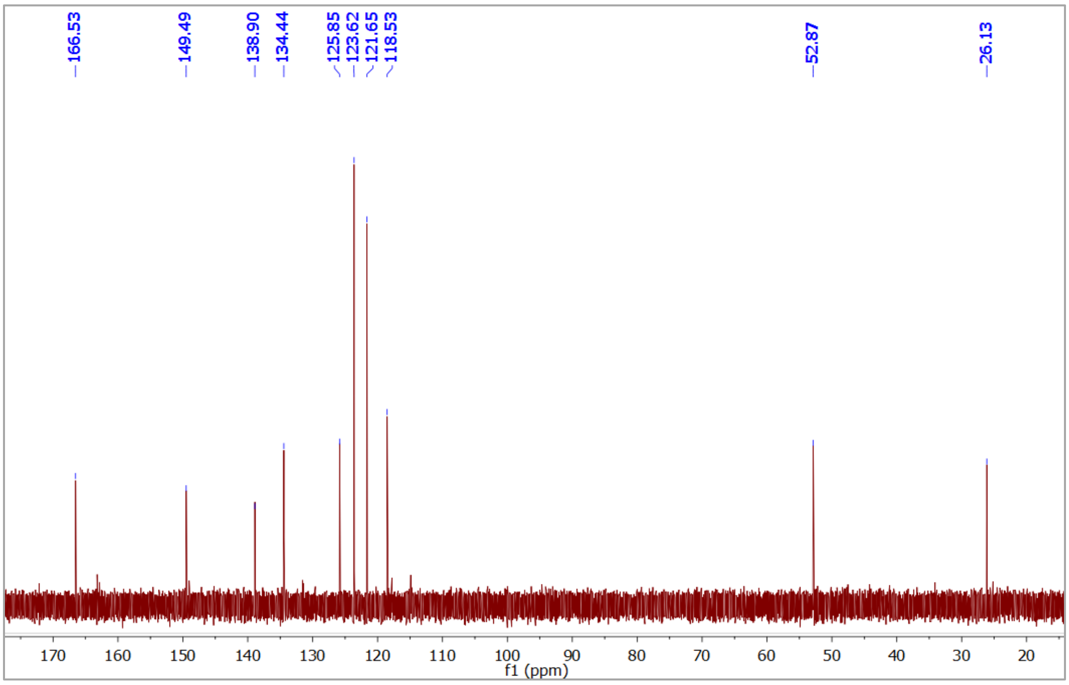


**Figure S4**. $^{13}$C NMR spectrum of **Azo-DD-his** (**5**).

**$^{13}$C NMR (101 MHz, Deuterium Oxide) δ:** 166.5, 149.5, 138.9, 134.4, 125.9, 123.6, 121.7, 118.5, 52.9, 26.1.

**HRMS m/z (ESI)**: $C_{24}H_{26}N_{10}O_2$ $[M+H]^+$, calculated: 487.2318, found: 487.2319.

**Azo4F-LL/DD-his** (**15** or **16**) were synthesized according to the route depicted in Scheme S2. Compounds **6** and **7** were synthesized according to a previously published procedure.[2]

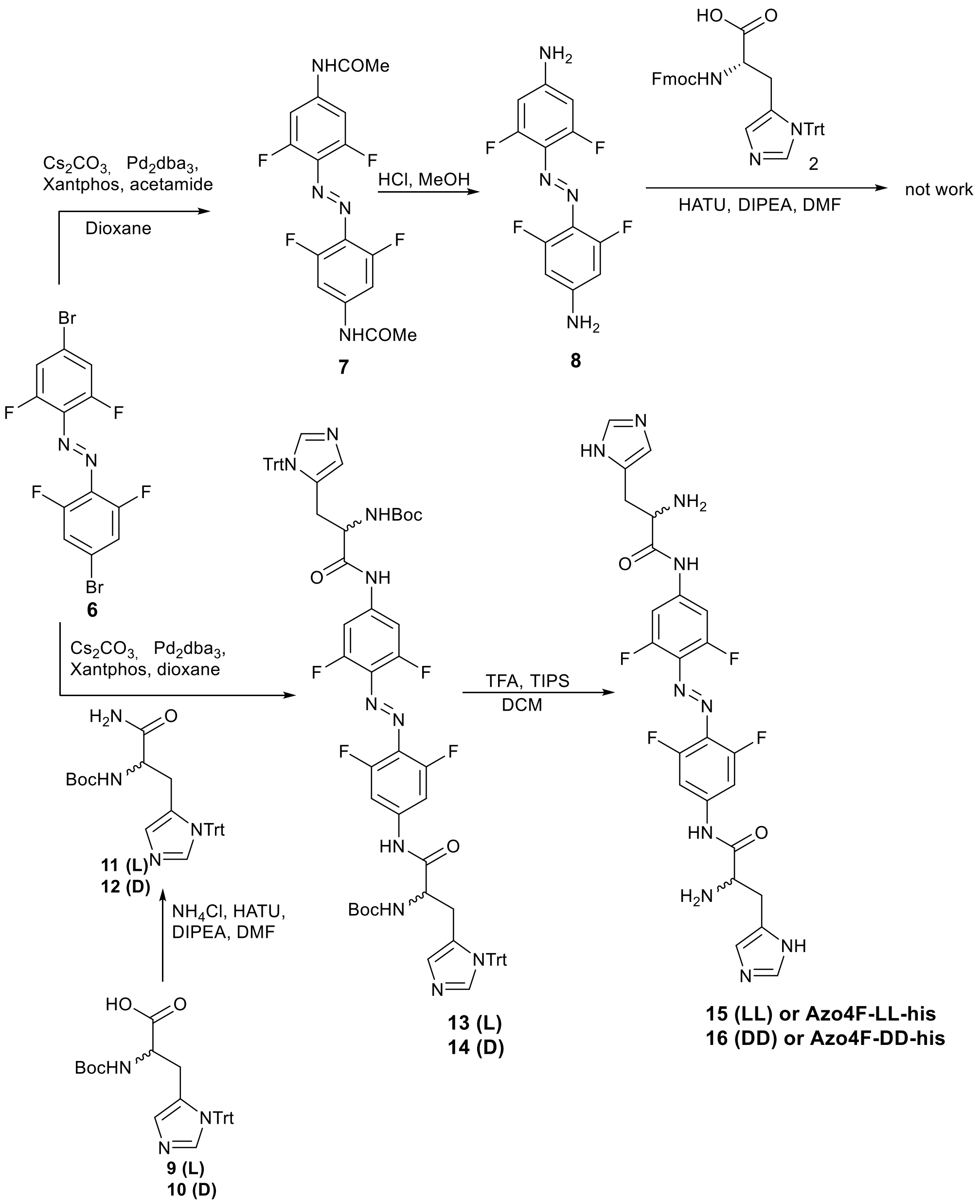


**Scheme S2**. Synthetic route of **Azo4F-LL-his** (**15**) and **Azo4F-DD-his** (**16**).

Synthesis of **8**

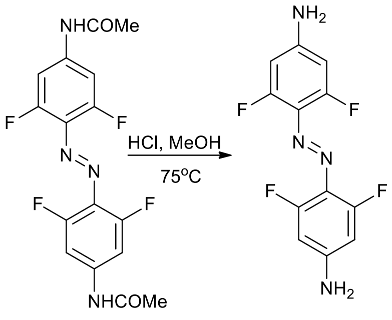


Into a solution of **7** (0.1 g, 0.27 mmol, 1 equiv.) in methanol (15 mL), 12 mL of 6 M HCl was added and the mixture was heated at 75 °C for 6 h. The solvent was evaporated and the residues were diluted with water (50 mL) and slowly neutralized by the addition of 2 M NaOH. The formed solid was filtered, washed with water and dried. The crude product was purified by column chromatography on $SiO_2$ using a gradient from EtOAc/hexane (1/1) to EtOAc and a product as an orange powder was obtained (37 mg, 48%).

**$^1$H NMR (400 MHz, DMSO-*d*$_6$) δ:** 6.27 – 6.20 (m, 4H), 3.78 (bs, 4H).

**HRMS m/z (ESI):** $C_{12}H_8F_4N_4$: $[M+H]^+$, calculated: 285.0763, found: 285.0760

**Synthesis of 11 and 12**

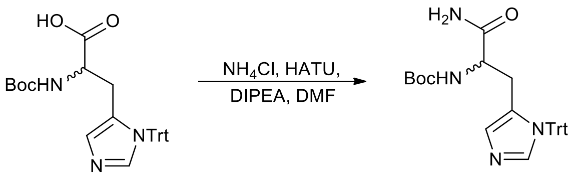


Into a solution **9** (or **10**) (0.3 g, 0.6 mmol, 1 eq.) in DMF (10 mL), DIPEA (0.18 g, 1.4 mmol, 2.4 eq.) and HATU (0.27 g, 0.7 mmol, 1.2 eq.) were added. After 30 s, ammonium chloride (0.1 g, 1.8 mmol, 3 eq.) was further added into the reaction mixture that was stirred for 2 hs at room temperature. Then, the mixture was diluted with water and extracted with EtOAc (3 x 50 mL). The combined organic phases were dried over $MgSO_4$, filtered, and concentrated under reduced pressure. The crude product was purified by column chromatography on $SiO_2$ gradient from DCM to 90/10 DCM/MeOH to give the product as a white powder.

**$^1$H NMR (601 MHz, Acetone-d6) δ:** 7.43 – 7.36 (m, 9H), 7.31 (s, 1H), 7.18 – 7.10 (m, 6H), 6.95 (s, 1H, CON**H**H), 6.69 (s, 1H, CONH**H**), 6.47 (d, J = 7.8 Hz, 1H, N**H**), 6.39 (s, 1H), 4.36 – 4.26 (m, 1H), 2.93 – 2.85 (m, 2H), 1.39 (s, 9H).

**HRMS m/z (ESI):** $C_{30}H_{32}N_4O_3$ $[M+H]^+$, calculated: 497.2553, found: 497.2553.

**Synthesis of 15 and 16**

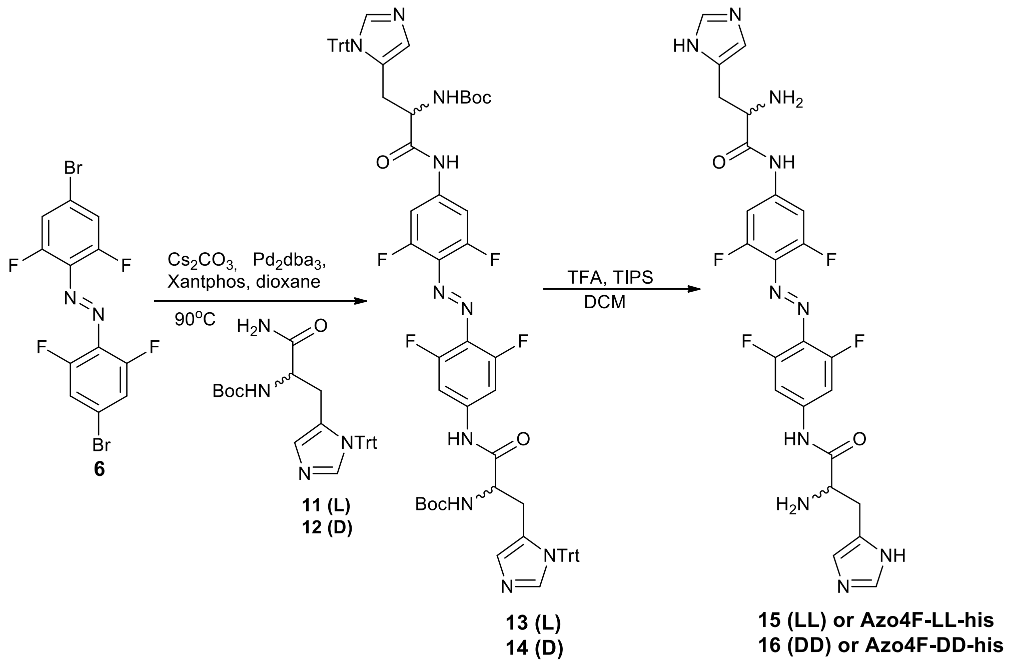


A flask was filled with **6** (40 mg, 0.1 mmol, 1 eq.), **11** (or **12**) (0.19 g, 0.4 mmol, 4 eq.), $Cs_2CO_3$ (0.11 g, 0.3 mmol, 3.5 eq.) and dioxane (4 mL). The resulting mixture was cooled down to 0 °C and $Pd_2dba_3$ (4.5 mg, 0.005 mmol) along with 4,5-Bis(diphenylphosphino)-9,9-dimethylxanthene (11 mg, 0.019 mmol) were added on the top of the frozen solution. Then, the solution was allowed to reach the room temperature under argon and successively heated at 90 °C for 2 h. The mixture was diluted with ethyl acetate and washed with brine. The two phases were separated and the organic phase was dried over $MgSO_4$, filtered, and concentrated under vacuum. Without further purification, Boc and Trt protection groups were removed by adding the obtained solid into the mixture of: 15 mL of DCM, 0.4 mL of TFA and 0.1 mL of TIPS. The resulting mixture was stirred for 2 h and then concentrated under reduced pressure. The crude product was purified by HPLC using a gradient from water to water/MeCN (90/10) in 25 minutes. **15**, **16** were obtained as orange powders (**15**: 39 mg, 38%, **16**: 37 mg, 36%).

Spectral data for **Azo4F-LL-his** (**15**):

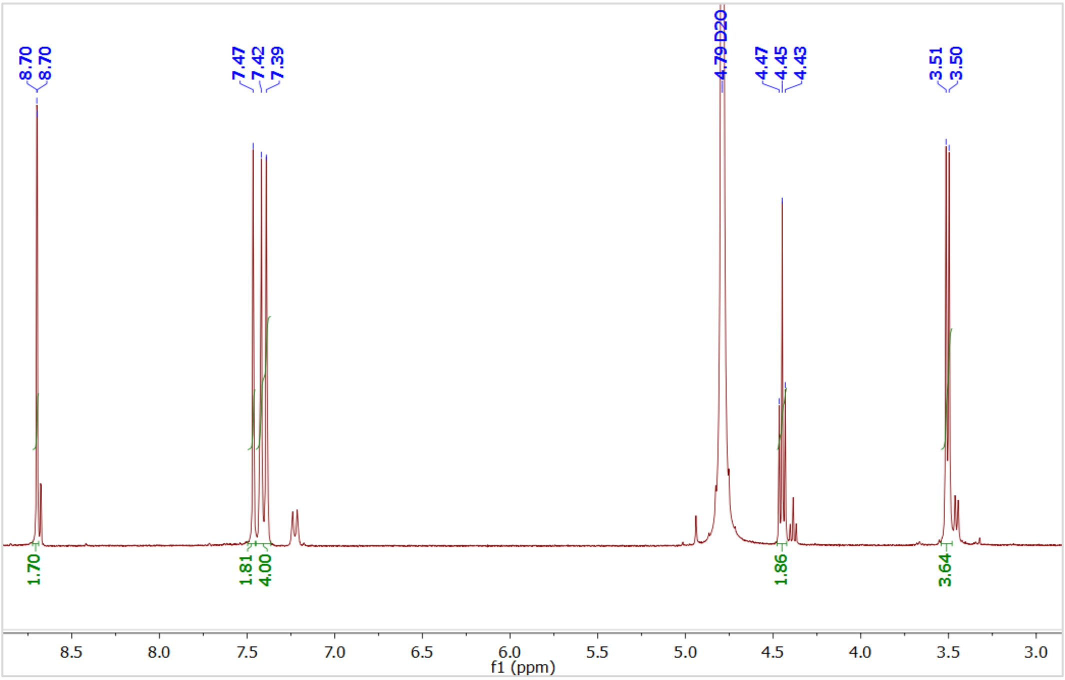


**Figure S5**. $^{1}$H NMR spectrum of **Azo4F-LL-his** (**15**).

**$^{1}$H NMR (400 MHz, Deuterium Oxide) δ:** 8.70 (d, *J* = 1.2 Hz, 2H), 7.53 (s, 1H), 7.44 – 7.37 (m, 4H), 4.45 (t, *J* = 6.9 Hz, 2H), 3.51 (d, *J* = 6.9 Hz, 4H).

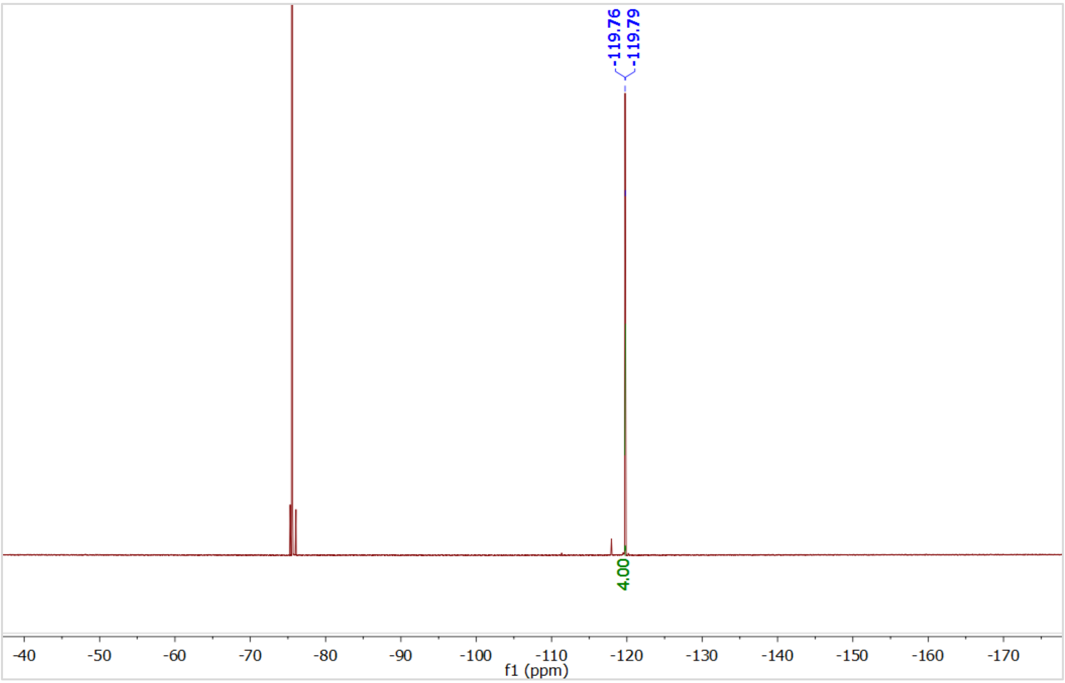


**Figure S6**. $^{19}$F NMR spectrum of **Azo4F-LL-his** (**15**).

**$^{19}$F NMR (376 MHz, Deuterium Oxide) δ:** -119.76 (m).

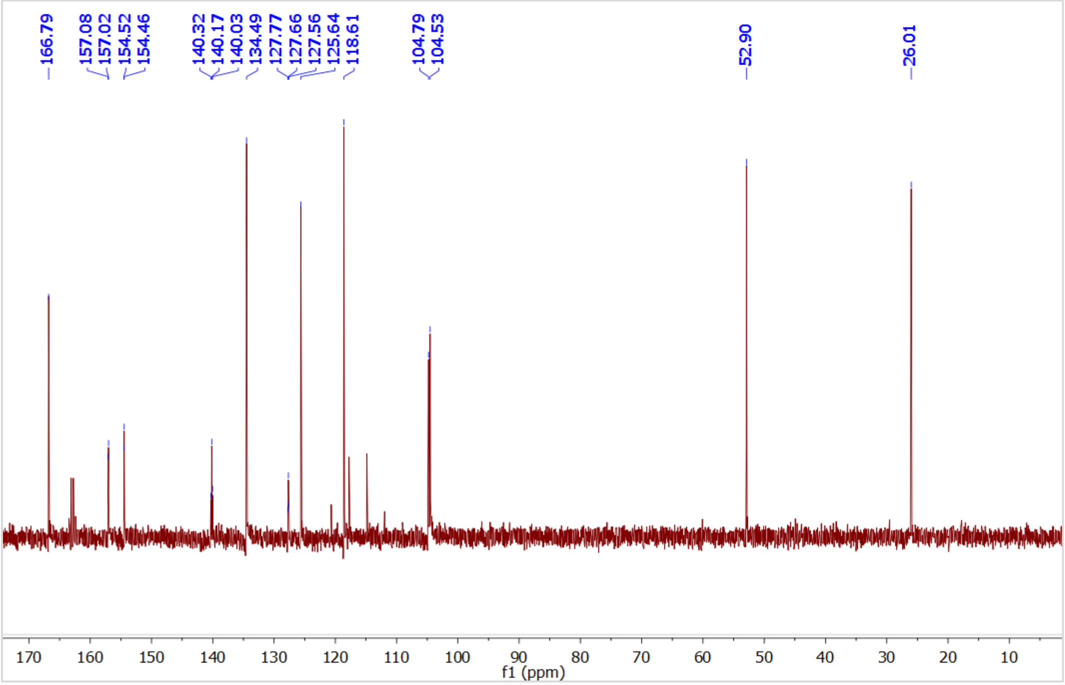


**Figure S7**. $^{13}$C NMR spectrum of **Azo4F-LL-his** (**15**).

**$^{13}$C NMR (101 MHz, Deuterium Oxide) δ:** 166.8, 155.8 (dd, $^{1}J_{CF}$ = 257.2, $^{3}J_{CF}$ = 6.2 Hz), 140.2 (t, $^{3}J_{CF}$ = 14.2 Hz), 134.5, 127.7 (t, $^{3}J_{CF}$ = 10.4 Hz), 125.6, 118.6, 104.7 (d, $^{2}J_{CF}$ = 26.2 Hz), 52.9, 26.0.

**HRMS m/z (ESI)**: $C_{24}H_{22}F_4N_{10}O_2$ $[M+H]^+$, calculated: 559.1942, found: 559.1942.

Spectral data for **Azo4F-DD-his** (**16**):

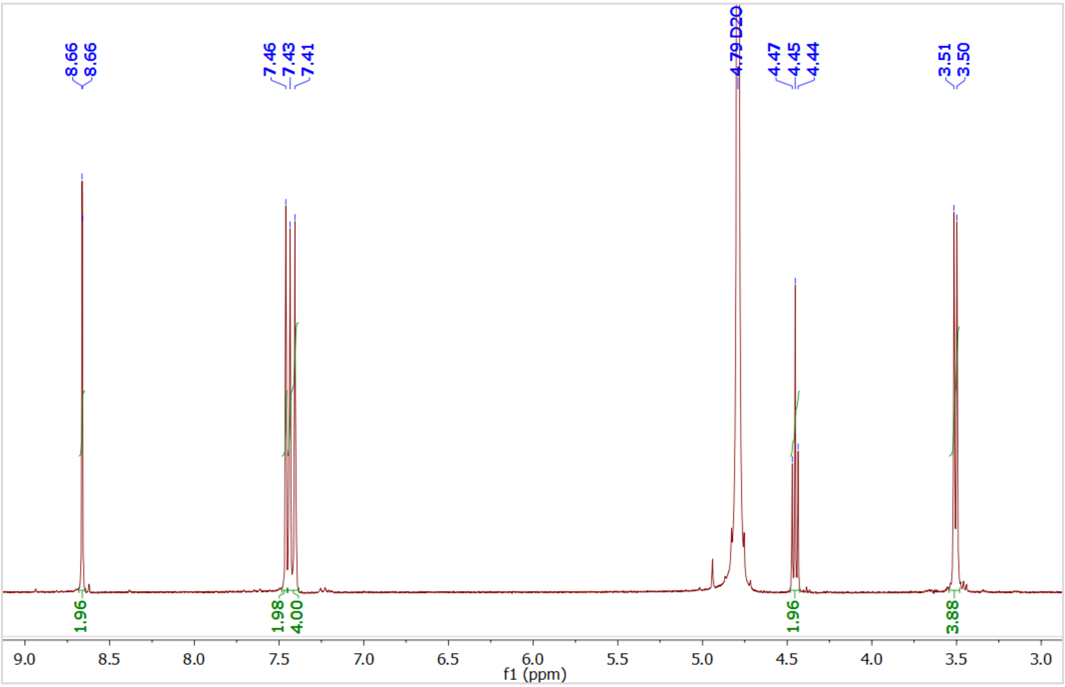


**Figure S8**. $^{1}$H NMR spectrum of **Azo4F-DD-his** (**16**).

**$^{1}H$ NMR (400 MHz, Deuterium Oxide) δ:** 8.66 (d, $J$ = 1.2 Hz, 2H), 7.46 (s, 1H), 7.45 – 7.39 (m, 4H), 4.45 (t, $J$ = 6.9 Hz, 2H), 3.51 (d, $J$ = 6.9 Hz, 4H).

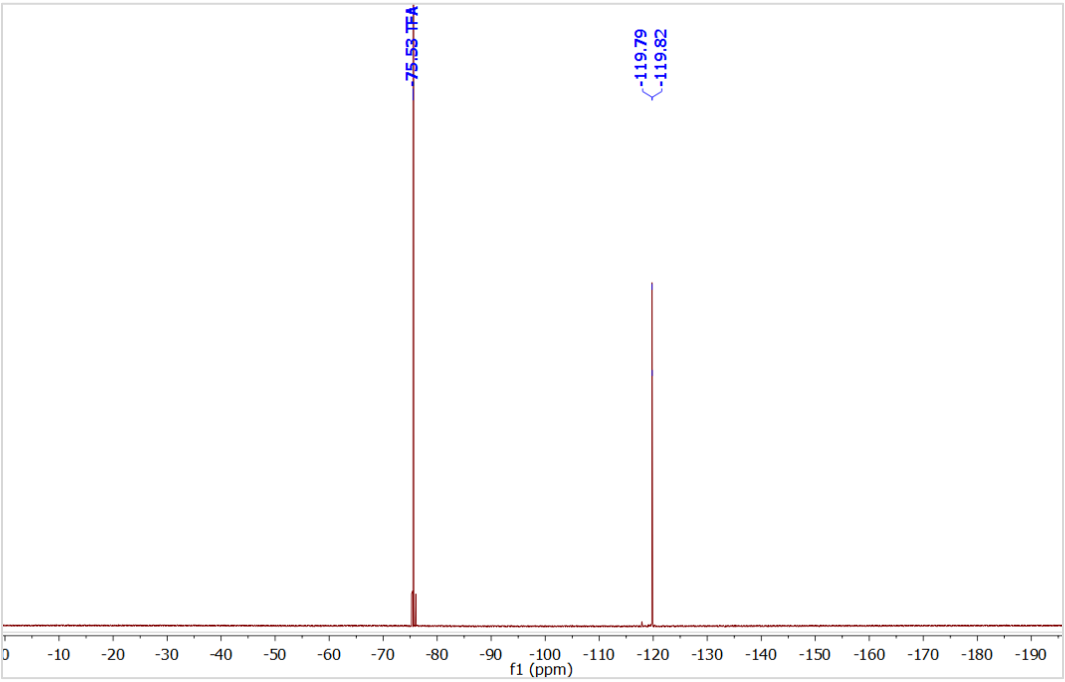


**Figure S9**. $^{19}F$ NMR spectrum of **Azo4F-DD-his** (**16**).

**$^{19}F$ NMR (376 MHz, Deuterium Oxide) δ:** -119.80 (m).

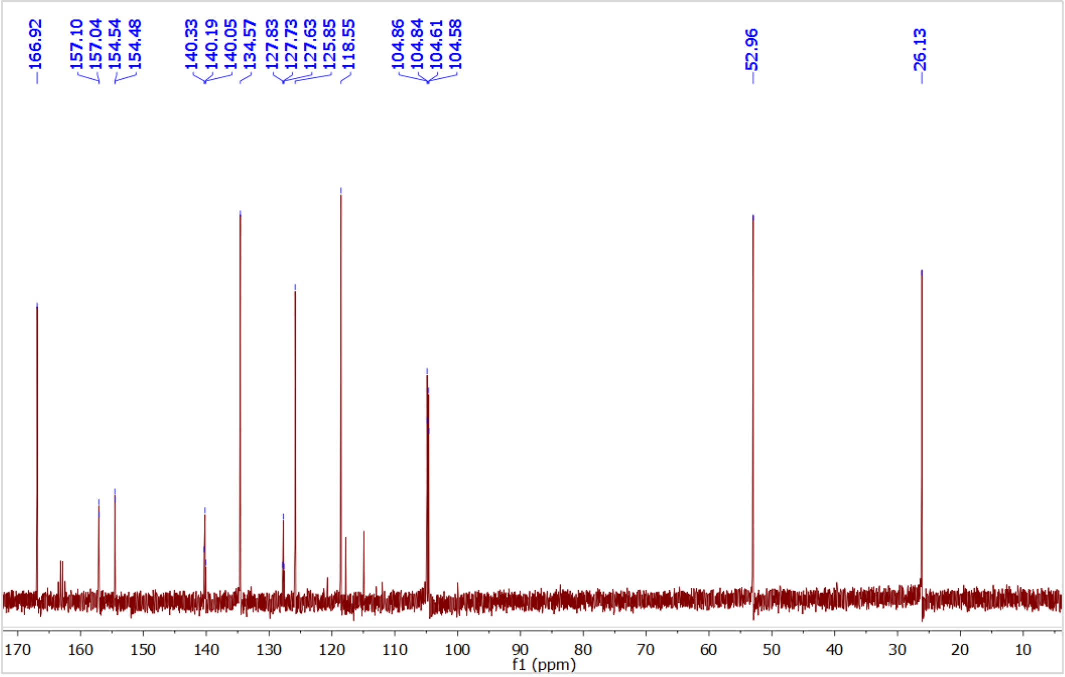


**Figure S10**. $^{13}C$ NMR spectrum of **Azo4F-DD-his** (**16**).

**$^{13}C$ NMR (101 MHz, Deuterium Oxide) δ:** 166.9, 155.8 (dd, $^{1}J_{CF}$ = 257.3, $^{3}J_{CF}$ = 6.1 Hz), 140.2 (t, $^{3}J_{CF}$ = 14.1 Hz), 134.6, 127.7 (t, $^{3}J_{CF}$ = 10.0 Hz), 125.9, 118.6, 104.7 (dd, $^{2}J_{CF}$ = 25.8, $^{4}J_{CF}$ =2.6 Hz), 53.0, 26.1.

**HRMS m/z (ESI)**: $C_{24}H_{22}F_{4}N_{10}O_{2}$ $[M+H]^{+}$, calculated: 559.1942, found: 559.1942.

# PHOTOISOMERIZATION STUDIES

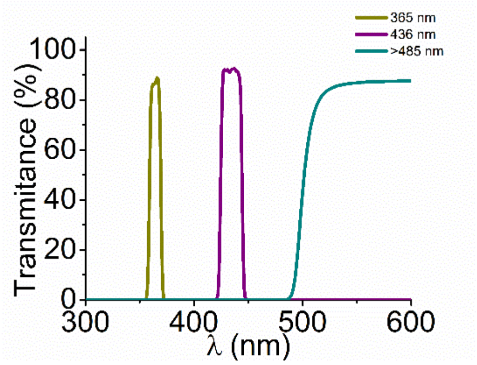


**Figure S11.** Transmission spectra of the filters used for irradiation.

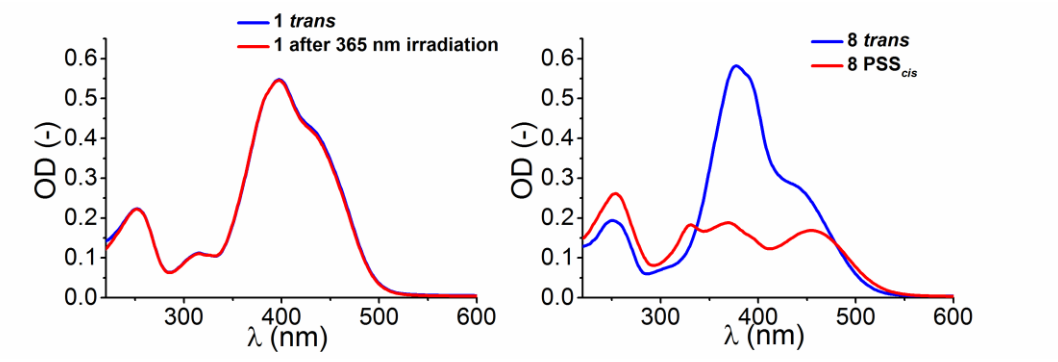


**Figure S12.** UV-Vis absorption spectra of **1** and **8** in methanol (20 μM). Red line corresponds to $PSS_{cis}$ state after 365 nm light irradiation.

The composition of the photostationary state was determined by HPLC for compound **Azo-DD-his** (Figure S13) or by $^1$H NMR for compound **Azo4F-LL-his** (Figure 1C).

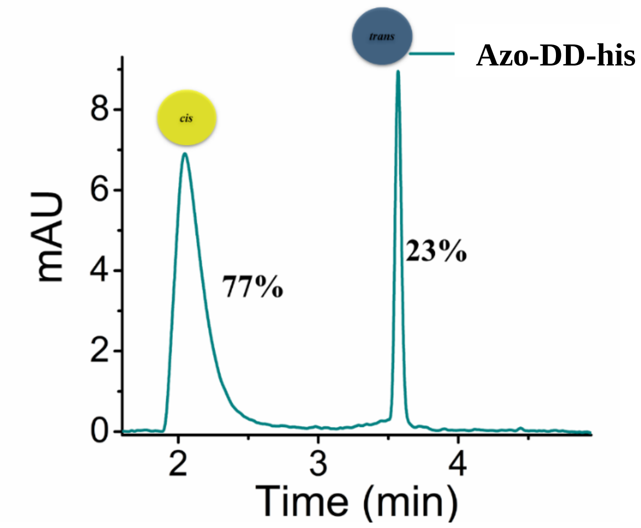


**Figure S13.** Chromatogram of **Azo-DD-his** after irradiation with UV light at 365 nm. The composition of $PSS_{cis}$ was found to be 77/23% of *cis/trans*.

**Table S1.** Spectroscopic characterization of the studied azobenzene analogues in water.

| **Azo switch** | **Spectroscopic data[a]** | | | | **PSS** | | | $\tau_{1/2}$[e] |
|---|---|---|---|---|---|---|---|---|
| | ***trans*** | | ***cis*** | | | | | |
| | **$\pi\rightarrow\pi^*$** | **$n\rightarrow\pi^*$** | **$\pi\rightarrow\pi^*$** | **$n\rightarrow\pi^*$** | | | | |
| | $\lambda_{max}$ [nm] | | $\lambda_{max}$ [nm] | | 436 nm [% of trans] | 365 nm [% of cis] | >485 nm [% of cis] | [h] |
| **Azo-DD/LL-his** | 359 | -[b] | 321 | 436 | - | 77[c] | - | 0.10 |
| **Azo4F-DD/LL-his** | 352 | 441 | 313 | 429 | 57[d] | 87[d] | 78[d] | 47 |

[a] UV/Vis spectra of azobenzene derivatives were measured in water. [b] The n–π* peak positions were unable to be distinguished due to overlapping with the tail of π – π * band. [c] Isomer content adopted from HPLC spectrum. [d] Isomer content adopted from $^1$H NMR spectra [e] The $t_{1/2}$ values at 25 °C were extrapolated from the kinetics data by using the Arrhenius equation (see Fig. S14-S15).

# THERMAL STABILITY

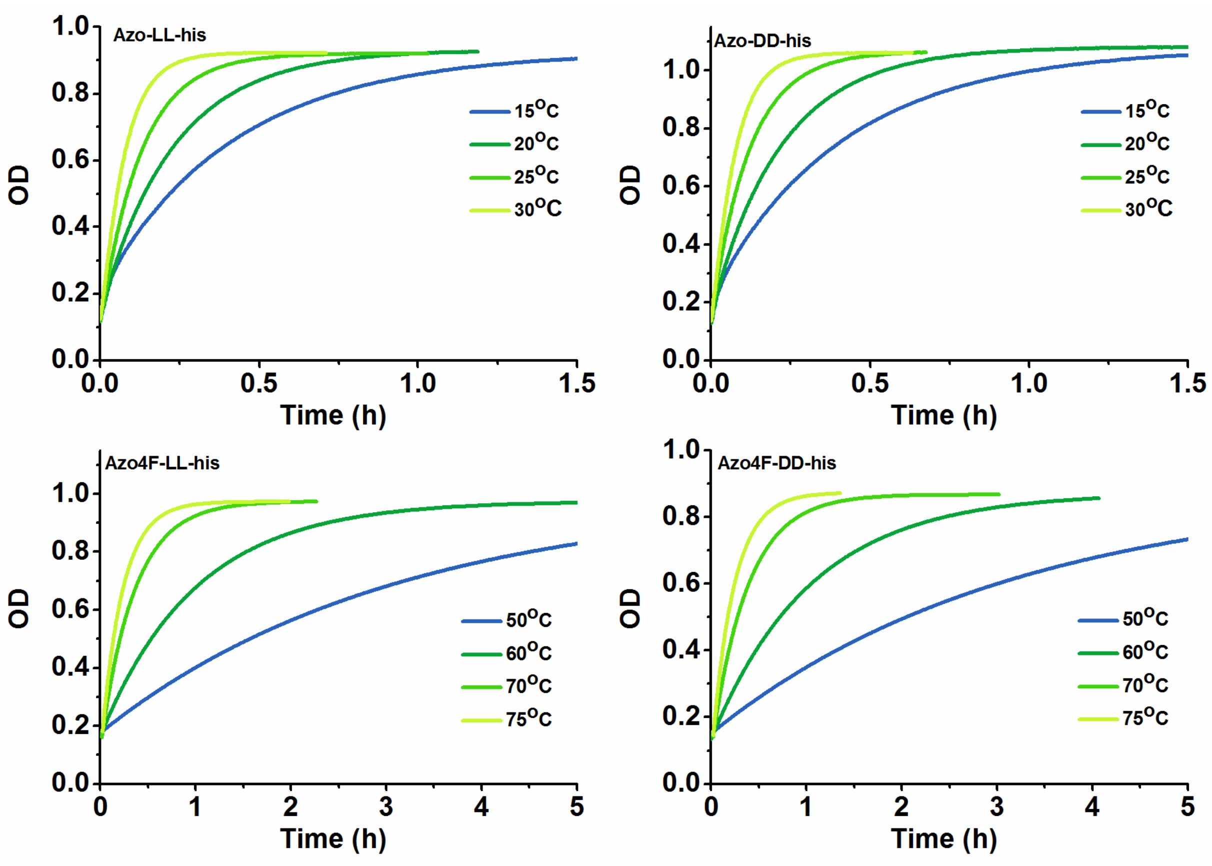


**Figure S14.** Thermal *cis-trans* isomerization of **Azo-LL/DD-his** and **Azo4F-LL/DD-his** in water at four different tempereatures: 15˚C, 20˚C, 25˚C and 30˚C for compounds **Azo-LL/DD-his** and 50˚C, 60˚C, 70˚C and 75˚C for compounds **Azo4F-LL/DD-his**.

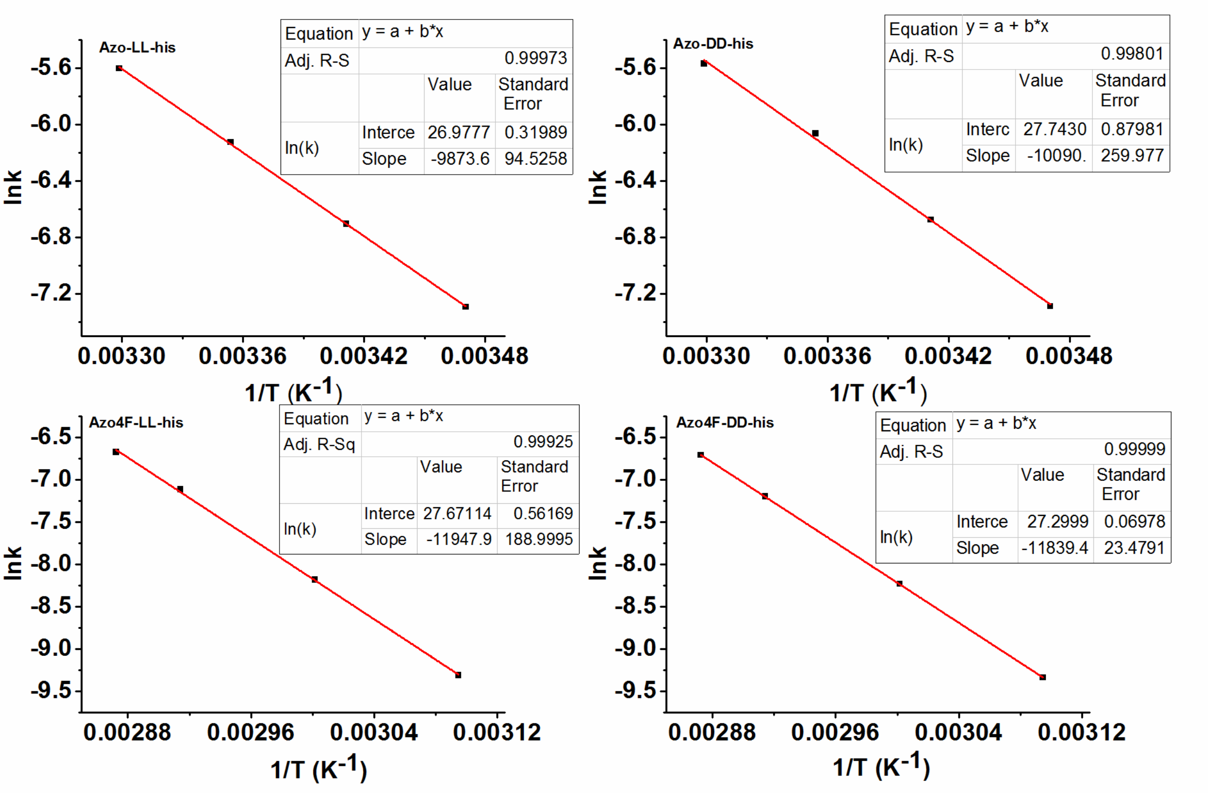


**Figure S15.** Arrhenius plots for **AzoLL/DD-his** and **Azo4F-LL/DD-his** in water. Rate constants for the *cis-trans* isomerization process, at different temperatures, were determined by the exponential curve fitting of the plots: absorbance *vs.* time. The slope is equal to: $-E_a/R$.

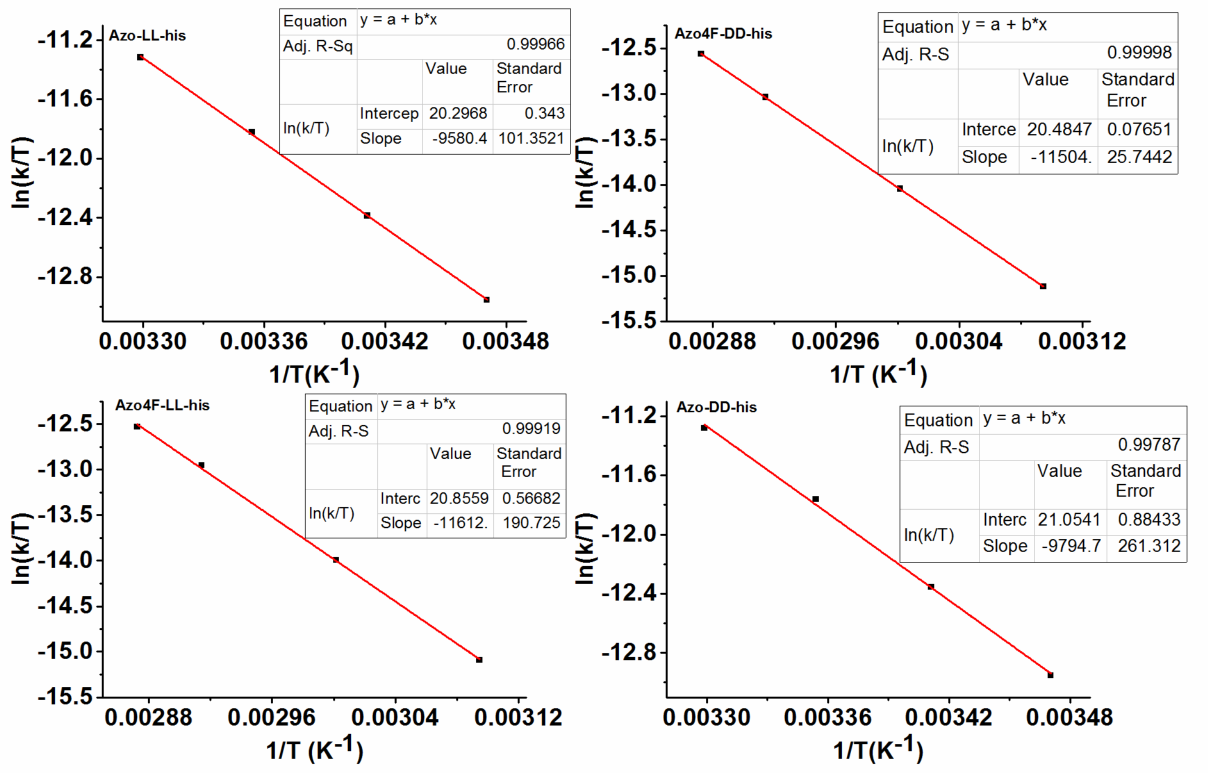


**Figure S16**. Eyring plots for **AzoLL/DD-his** and **Azo4F-LL/DD-his** in water. The slope is equal to: $-\Delta H^{\ddagger}/R$ and the intercept is equal to: $\ln((\kappa k_B)/h) + \Delta S^{\ddagger}/R$.

**Table S2.** Isomerization rates $k_{Z\rightarrow E}$ (at 298 K), thermal half-life $\tau_{1/2}$ (at 298 K), Arrhenius prefactor A, activation energies $E_a$, as well as Eyring activation free energies $\Delta G^{\ddagger}$, enthalpies $\Delta H^{\ddagger}$ and entropies $\Delta S^{\ddagger}$ for compounds **AzoLL/DD-his** and **Azo4F-LL/DD-his** in water.

| Cmpd | $k_{cis\rightarrow trans}^{[a]}$ [$s^{-1}$] | $lnk_{cis\rightarrow trans}$ | $\tau_{1/2}$ [h] | A[c] [$s^{-1}$] | Ea [$\frac{kJ}{mol}$] | $\Delta H^{\ddagger}$ [$\frac{kJ}{mol}$] | $\Delta S^{\ddagger}$ [$\frac{J}{mol \times K}$] | $\Delta G^{\ddagger}$ [$\frac{kJ}{mol}$] |
|---|---|---|---|---|---|---|---|---|
| **Azo-LL-his** | 2190 ± 219 | -6.10 ± 0.01 | 0.10 ± 0.01 | 5.2 ± 1.4 | 82.1 ± 0.8 | 80.2 ± 0.8 | -26.8± 1.2 | 88.1 ± 0.8 |
| **Azo-DD-his** | 2330 ± 233 | -6.10 ± 0.01 | 0.10 ± 0.01 | 11.2 ± 6.5 | 83.9 ± 2.2 | 81.4 ± 2.2 | -22.5 ± 0.9 | 88.1 ± 2.2 |
| **Azo4F-LL-his** | 4.1[b] ± 1.8 | -12.4 ± 0.6 | 47 ± 4.6 | 10.2 ± 1.9 | 99.3 ± 1.6 | 96.5 ± 1.6 | -24.1 ± 0.7 | 103.7 ± 1.6 |
| **Azo4F-DD-his** | 4.1[b] ± 0.4 | -12.4± 0.1 | 47 ± 4.6 | 7.2 ± 0.5 | 98.4 ± 0.2 | 95.6 ± 0.2 | -27.2 ± 0.1 | 103.7 ± 0.2 |

[a] $10^{-6}$ at 298 K. [b] estimated from Arrhenius equation at 298 K. [c] $10^{11}$

# G4 STUDIES

## DNA CHARACTERIZATION

**Oligonucleotide annealing**. G4 stock solutions ($c_{KCl}$ = 100 mM, Tris buffer 10.0 mM, pH= 7.4) were heated up at 95 °C for 5 min and then let slowly cooled down to room temperature overnight. The sequence and topology of the G4s used in this study are described in Table S3.

**Table S3**. Oligonucleotides used in this study.

| Name | Sequence[a] | Length (bp) | Topology and description |
|---|---|---|---|
| **Tel-22** | AGGGTTAGGGTTAGGGTTAGGG | 22 | hybrid/antiparallel[4] |
| **Bom17** | GGTTAGGTTAGGTTAGG | 17 | Anti-parallel[5] |
| ***c-MYC* Pu22** | TGAGGGTGGGTAGGGTGGGTAA | 22 | parallel[6] |
| **Z-G4** | TGGTGGTGGTGGTTGTGGTGGTGGTGTT | 28 | left-handed[7] |
| **dsDNA** | CAATCGGATCGAATTCGATCCGATTG | 26 | double stranded[6] |

[a] Conventional 5' to 3' direction.

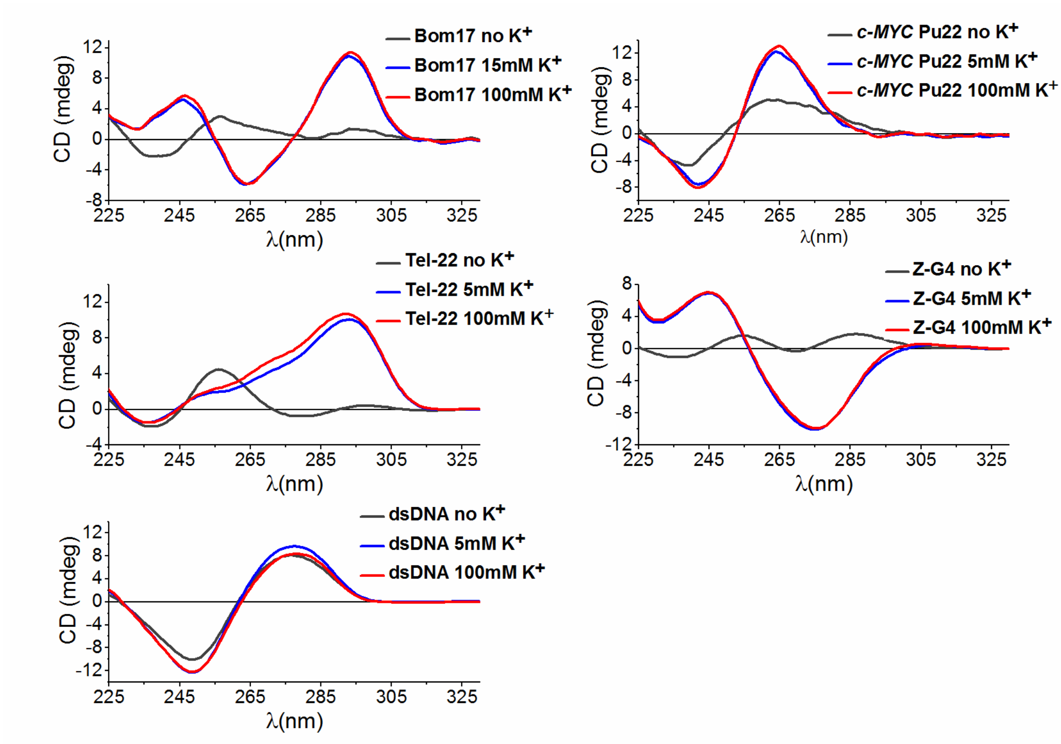


**Figure S17.** ECD spectra of the quadruplex and duplex DNA structures used in this study. Measurements were performed in 50 mM Tris-HCl (pH = 7.4) using 0, 5, 15 (Bom17) or 100 mM KCl.

# ECD spectra of Azo-LL/DD-his and Azo4F-LL/DD-his

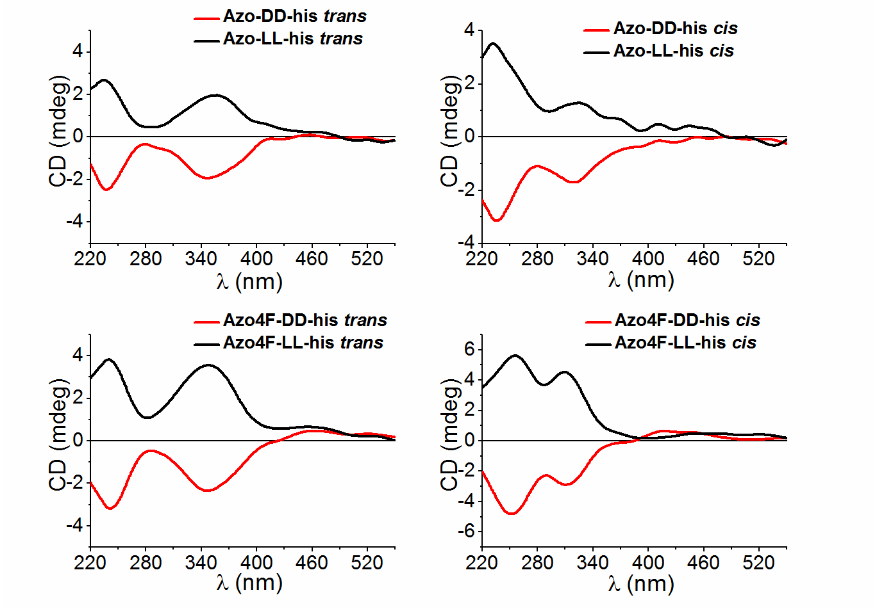


**Figure S18.** ECD spectra of the photochromes (30 µM) in water.

# MELTING STUDIES

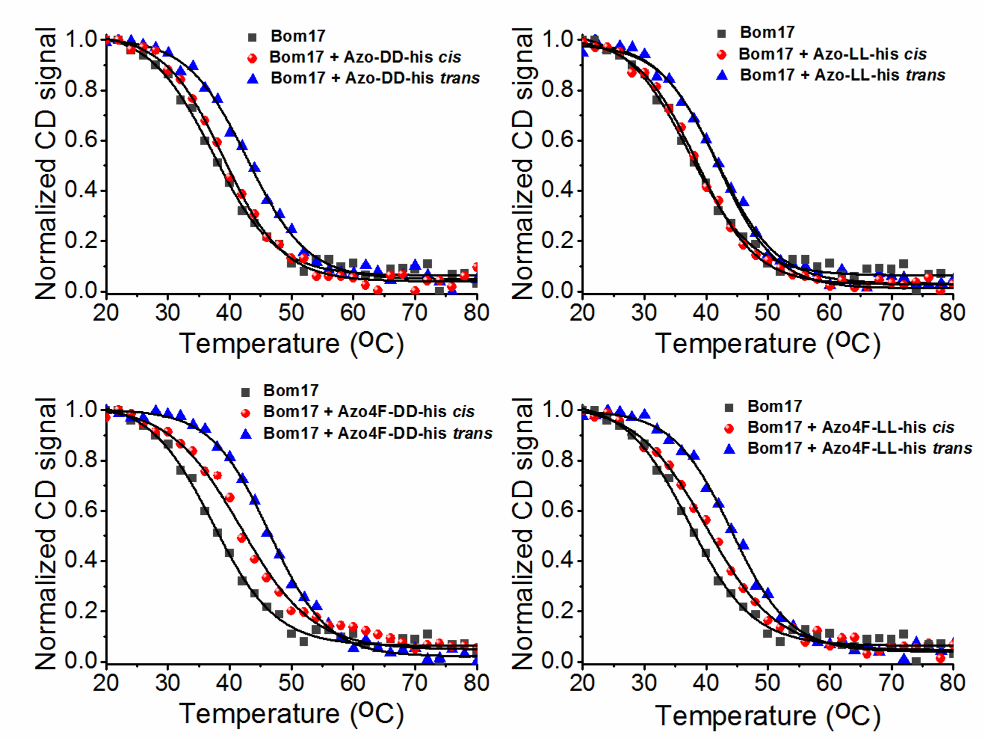


**Figure S19.** Melting curves for Bom17 ($K^+$) in the absence and presence of the photochromes in *trans* form or as *cis*-rich PSS ($C_{photochrome}$= 8.0 µM, $C_{G4s}$ = 2.0 µM, $C_{KCl}$ = 15 mM, $C_{Tris}$ = 10 mM pH 7.4).

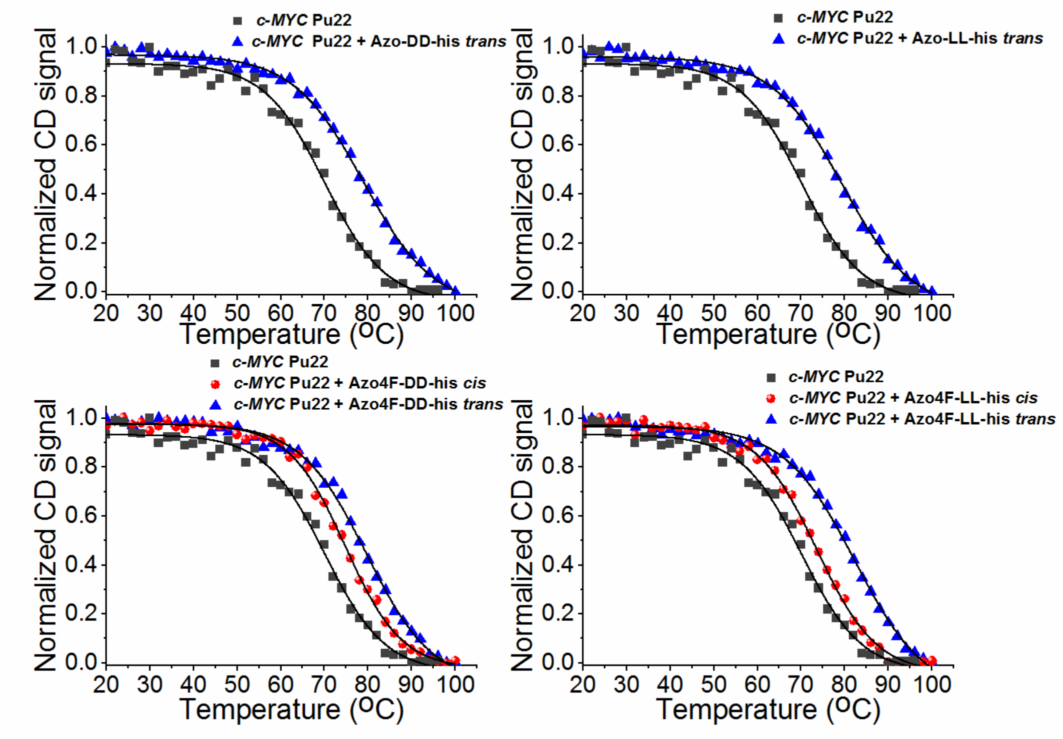


**Figure S20.** Melting curves for *c-MYC* Pu22 ($K^+$) in the absence and presence of the photochromes in *trans* form and, when possible, as *cis*-rich PSS ($C_{photochrome}$ = 8.0 µM, $C_{G4s}$ = 2.0 µM, $C_{KCl}$ = 5 mM, $C_{Tris}$ = 10 mM pH 7.4).

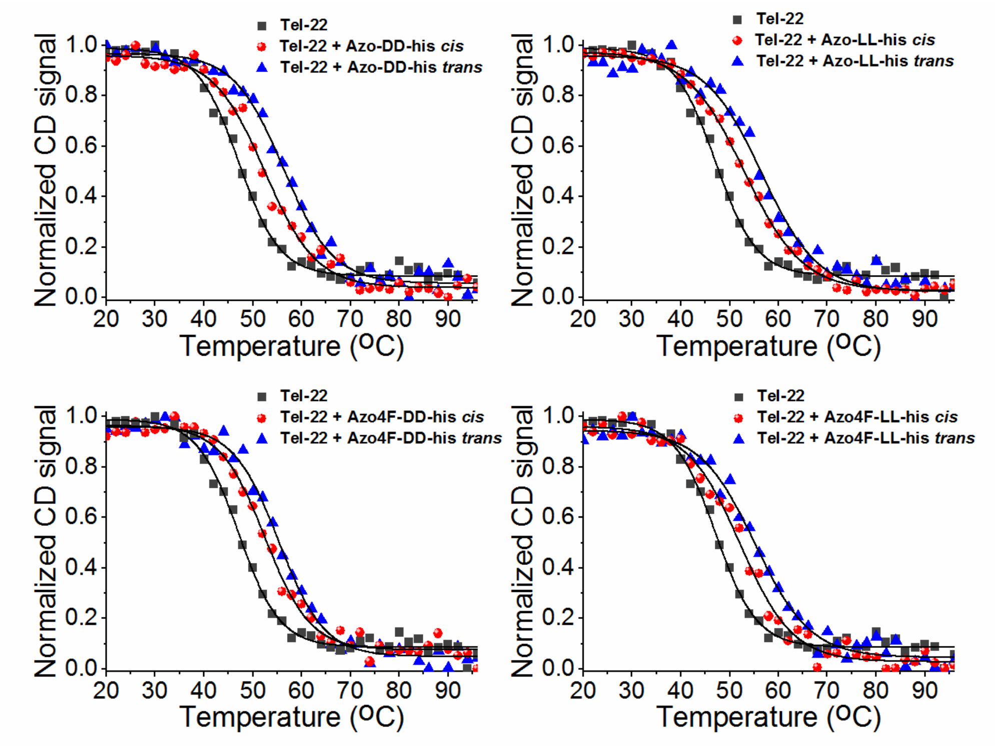


**Figure S21**. Melting curves for Tel22 ($K^+$) in the absence and presence of the photochromes in *trans* form or as *cis*-rich PSS ($C_{photochrome}$= 8.0 μM, $C_{G4s}$ = 2.0 μM, $C_{KCl}$ = 5 mM, $C_{Tris}$ = 10 mM pH 7.4).

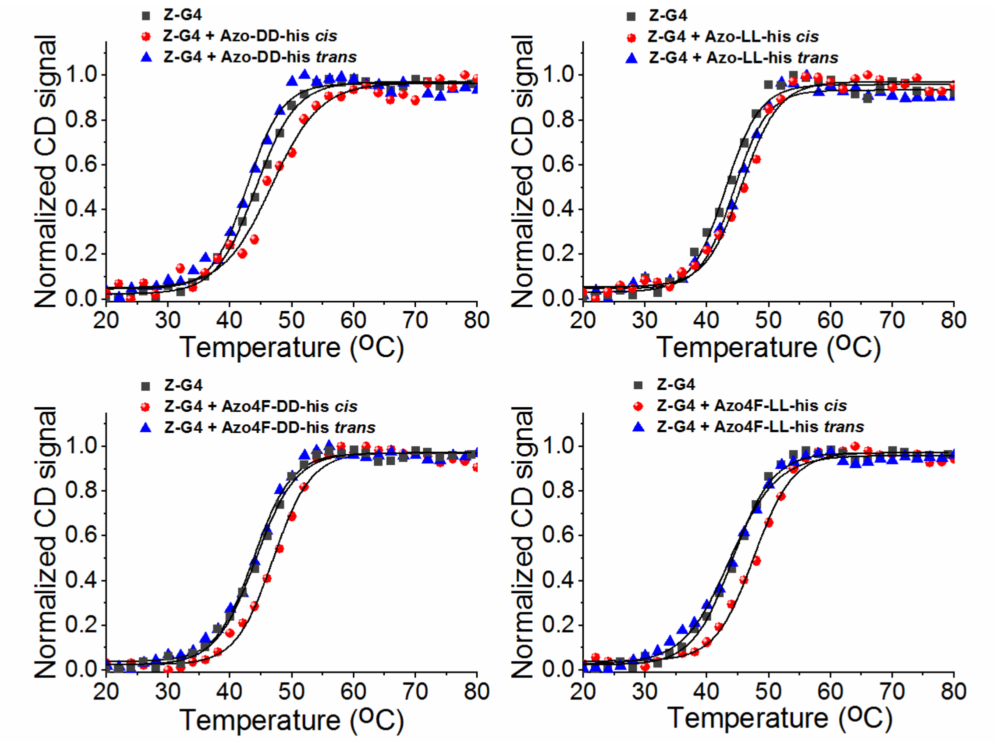


**Figure S22.** Melting curves for Z-G4 ($K^+$) in the absence and presence of the photochromes in *trans* form or as *cis*-rich PSS ($C_{photochrome}$= 8.0 μM, $C_{G4s}$ = 2.0 μM, $C_{KCl}$ = 5 mM, $C_{Tris}$ = 10 mM pH 7.4).

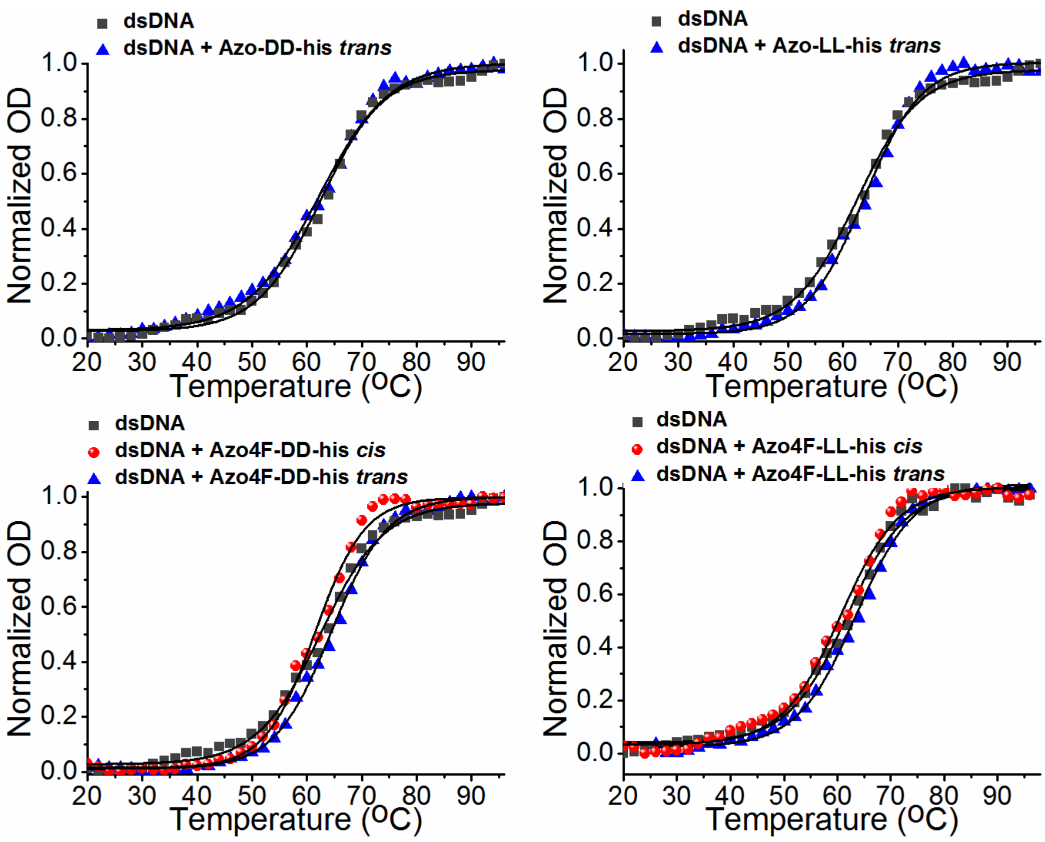


**Figure S23.** Melting curves for dsDNA in the absence and presence of the photochromes in *trans* form and, when possible, as *cis*-rich PSS ($C_{photochrome}$= 8.0 μM, $C_{ds\text{-}DNA}$ = 2.0 μM, $C_{KCl}$ = 5 mM, $C_{Tris}$ = 10 mM pH 7.4).

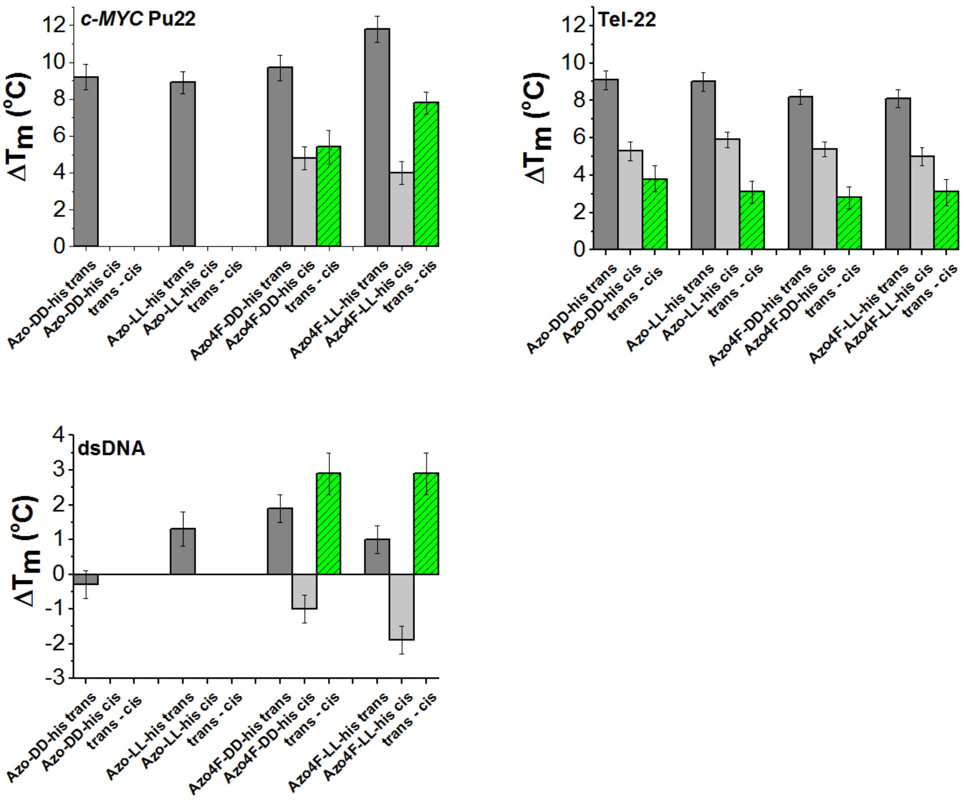


**Figure S24.** Induced thermal stabilization by **Azo-DD/LL-his** and **Azo4F-DD/LL-his** (*trans* and, when possible, *cis*-rich PSS) on *c-MYC* Pu22, Tel-22 and dsDNA. Results are presented as an average of three independent experiments. The concentration of KCl in Tris-HCl buffer (10 mM) was 5 mM.

**Table S4.** Melting temperature (in °C) of Bom17, *c-MYC* Pu22, Tel-22, Z-G4 and dsDNA (all 2 μM), in the presence of the photochromes (8 μM) in *trans* form and, when possible, *cis*-rich PSS.

| **System** | | **Bom17** | | | ***c-MYC* Pu22** | | | **Tel-22** | | | **Z-G4** | | | **dsDNA** | |
|---|---|---|---|---|---|---|---|---|---|---|---|---|---|---|---|
| G4/duplex alone | | **37.1** | | | **70.1** | | | **47.2** | | | **44.3** | | | **62.5** | |
| | $T_m^{trans}$ | $T_m^{cis}$ | $\Delta T_m$ | $T_m^{trans}$ | $T_m^{cis}$ | $\Delta T_m$ | $T_m^{trans}$ | $T_m^{cis}$ | $\Delta T_m$ | $T_m^{trans}$ | $T_m^{cis}$ | $\Delta T_m$ | $T_m^{trans}$ | $T_m^{cis}$ | $\Delta T_m$ |
| **Azo-DD-his** | 41.8 | 38.8[a] | 3.0 | 79.3 | - | - | 56.3 | 52.5[a] | 3.8 | 43.4 | 46.6[a] | -3.2 | 62.2 | - | - |
| **Azo-LL-his** | 40.9 | 38.5[a] | 2.4 | 79.0 | - | - | 56.2 | 53.1[a] | 3.1 | 45.5 | 45.7[a] | -0.2 | 63.8 | - | - |
| **Azo4F-DD-his** | 46.1 | 41.8 | 4.3 | 79.8 | 74.4[a] | 5.4 | 55.4 | 52.6 | 2.8 | 43.9 | 46.3 | -2.4 | 64.4 | 61.5 | 2.9 |
| **Azo4F-LL-his** | 43.3 | 39.7 | 3.6 | 81.9 | 74.1[a] | 7.8 | 55.3 | 52.2 | 3.1 | 44.3 | 47.7 | -3.4 | 63.5 | 60.6 | 2.9 |

[a] The system was irradiated with UV light (365 nm) during the measurement.

## NMR STUDIES

**A)**

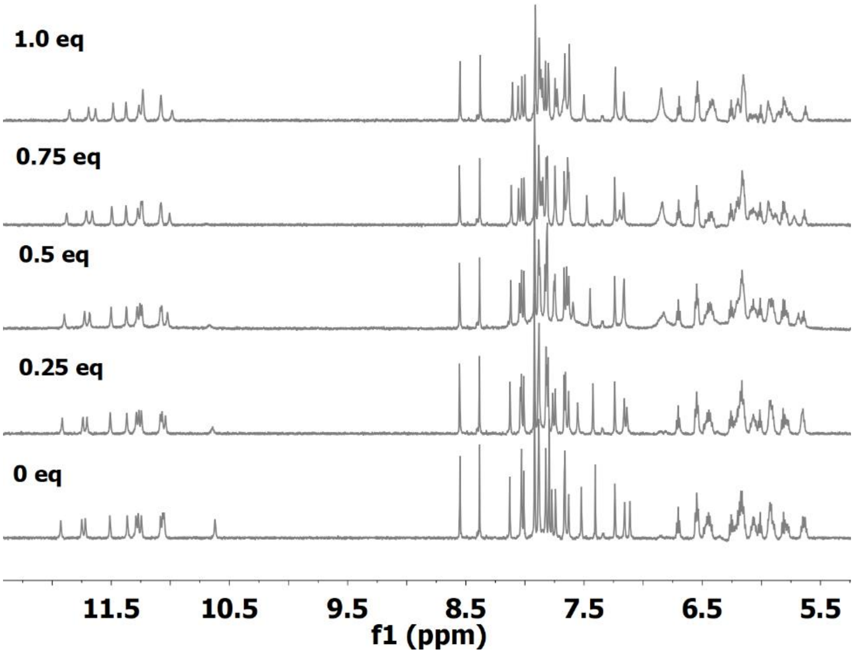


**B)**

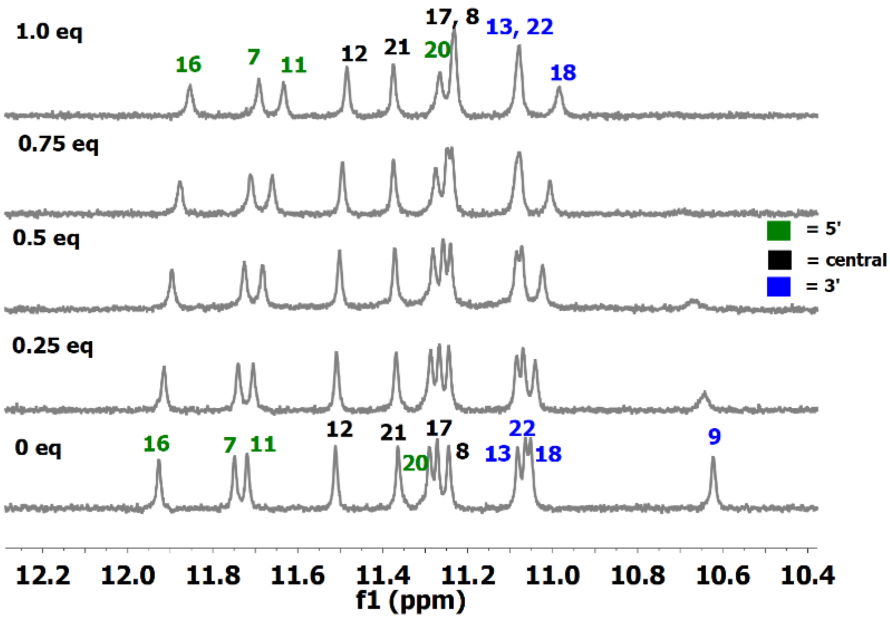


**Figure S25.** (A) $^{1}$H NMR spectra (imino/aromatic region) of *c-MYC* Pu22 titrated with **Azo4F-DD-his** at the *cis*-rich PSS. (B) $^{1}$H NMR spectra of the G-tetrad imino protons in the absence and presence of **Azo4F-DD-his** (*cis*-rich PSS). The imino protons of the 5′-end are colored coded in green, the central G-tetrad in black and the 3′-end in blue.

A)

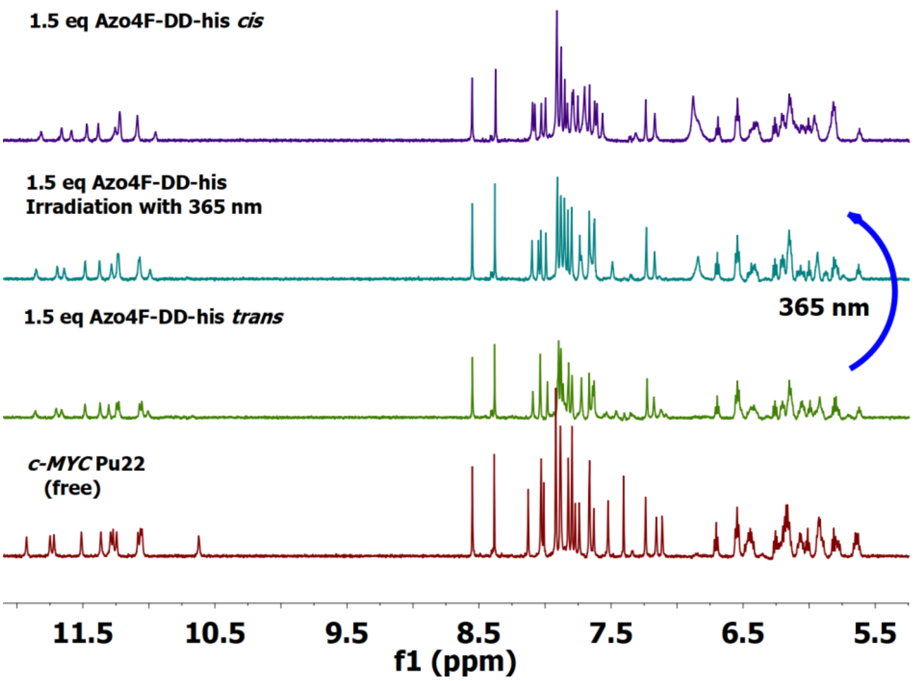


B)

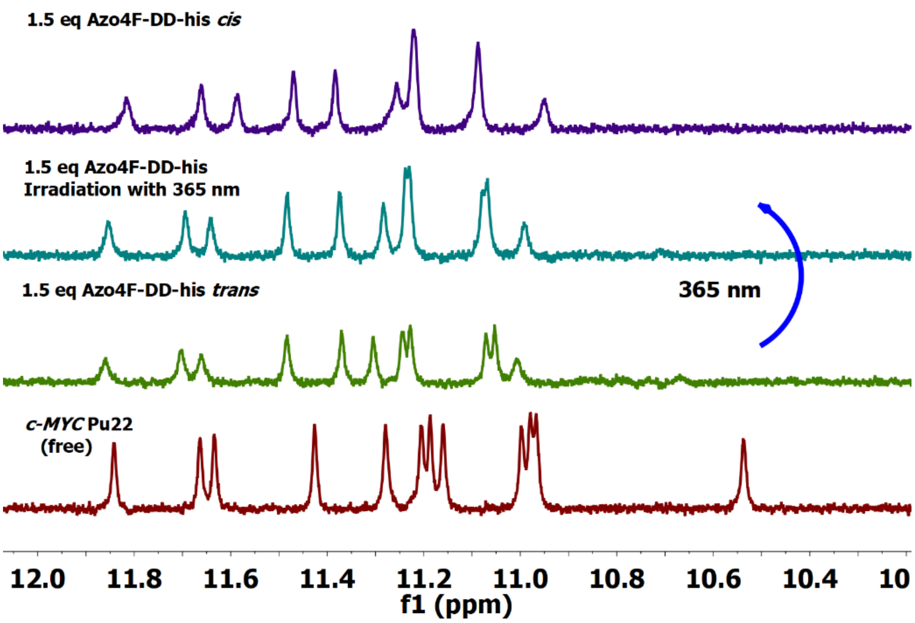


**Figure S26.** $^{1}$H NMR spectra (A) imino/aromatic region and (B) imino region) of *c-MYC* Pu22 with **Azo4F-DD-his** *trans* and **Azo4F-DD-his** at the *cis*-rich PSS. The $^{1}$H NMR spectrum, for the *in-situ* photoconversion of **Azo4F-LL-his** *trans* complexed with *c-MYC* Pu22, was recorded after UV light (365 nm) irradiation (*trans*-to- *cis*-rich PSS conversion) and clearly shows the reversibility of the process.

## ECD spectra of Azo-LL/DD-his complexed with duplex and quadruplexes

**Azo4F-DD/LL-his** under the following experimental conditions: (50 µM of azobenzene, 50 mM Tris-HCl pH = 7.4, 100 mM KCl) displayed intense induced circular dichroism band and we measured, therefore, the ECD spectra of only **Azo-LL/DD-his** complexed with G4 and duplex DNAs.

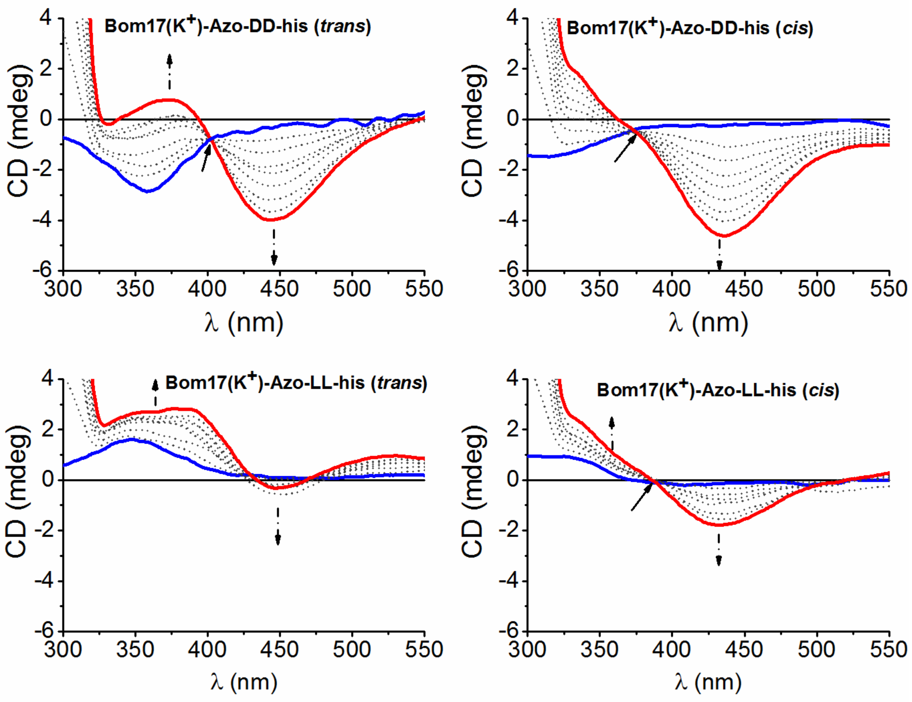


**Figure S27.** ECD spectra resulting from the complexation of **Azo-LL/DD-his** *trans* or *cis*-rich PSS with Bom17 ($K^+$). [**Azo-LL/DD-his**] = 50 µM and [Bom17 ($K^+$)] = from 0 to 20 µM. Blue line corresponds to the free ligand and the red line corresponds to the ligand complexed with Bom17.

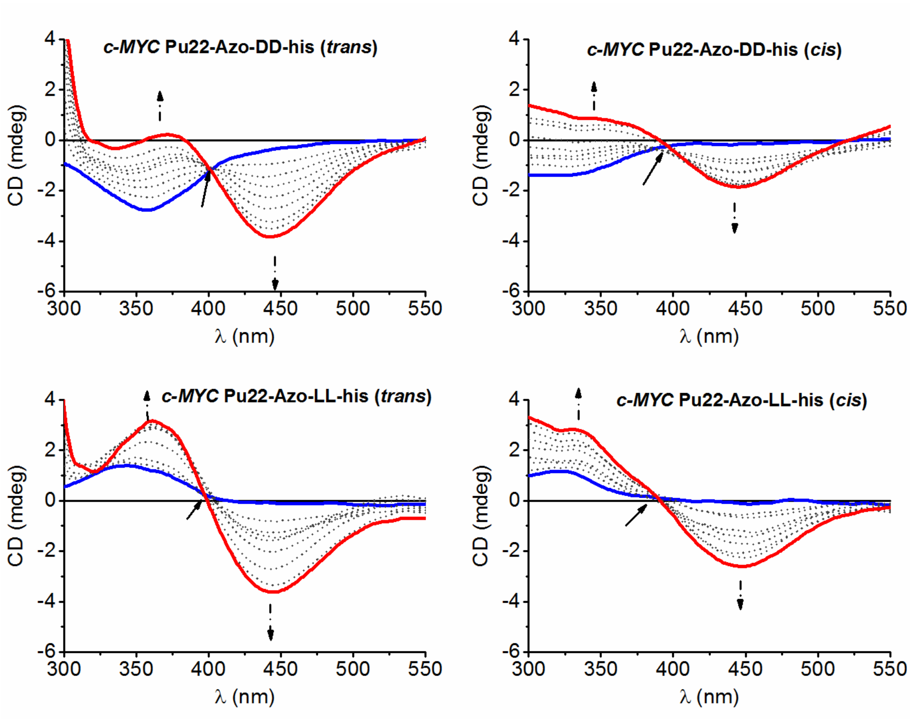


**Figure S28**. ECD spectra resulting from the complexation of **Azo-LL/DD-his** *trans* or *cis*-rich PSS with *c-MYC* Pu22 ($K^+$). [**Azo-LL/DD-his**] = 50 μM and [*c-MYC* Pu22 ($K^+$)] = from 0 to 20 μM. Blue line corresponds to the free ligand and the red line corresponds to the ligand complexed with *c-MYC* Pu22.

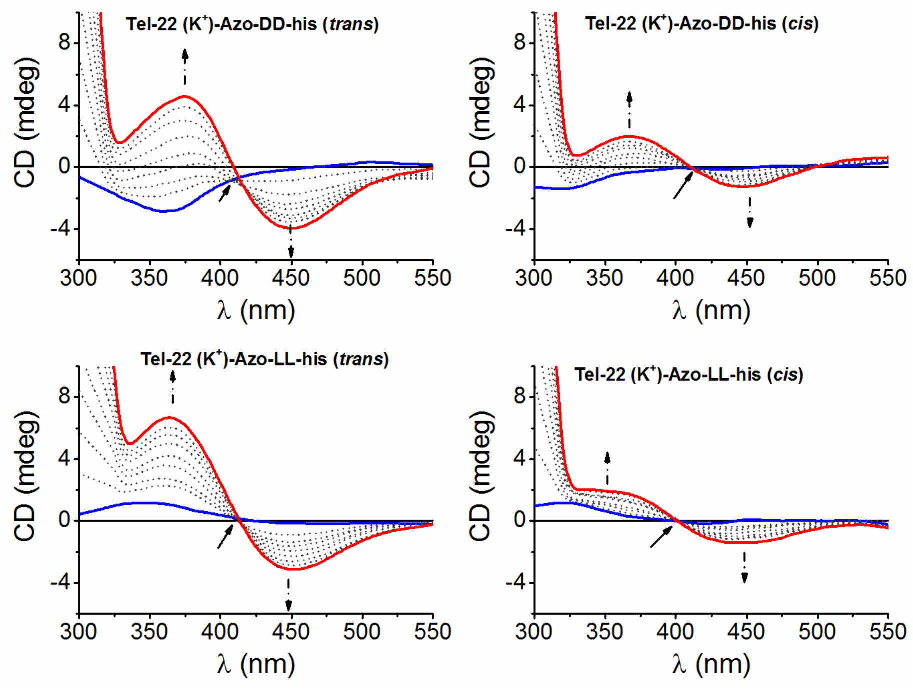


**Figure S29.** ECD spectra resulting from the complexation of **Azo-LL/DD-his** *trans* or *cis*-rich PSS with Tel-22 ($K^+$). [**Azo-LL/DD-his** *trans* or *cis*] = 50 μM and [Tel-22 ($K^+$)] = from 0 to 20 μM. Blue line corresponds to the free ligand and the red line corresponds to the ligand complexed with Tel-22.

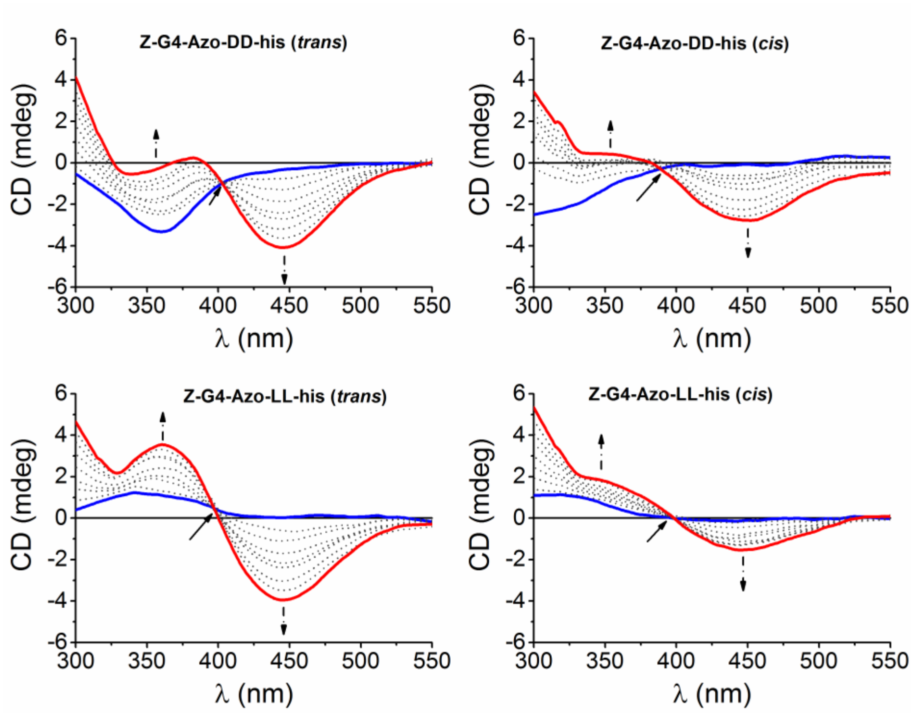


**Figure S30.** ECD spectra resulting from the complexation of **Azo-LL/DD-his** *trans* or *cis*-rich PSS with Z-G4. [**Azo-LL/DD-his**] = 50 μM and [Z-G4 ($K^+$)] = from 0 to 20 μM. Blue line corresponds to the free ligand and the red line corresponds to the ligand complexed with Z-G4.

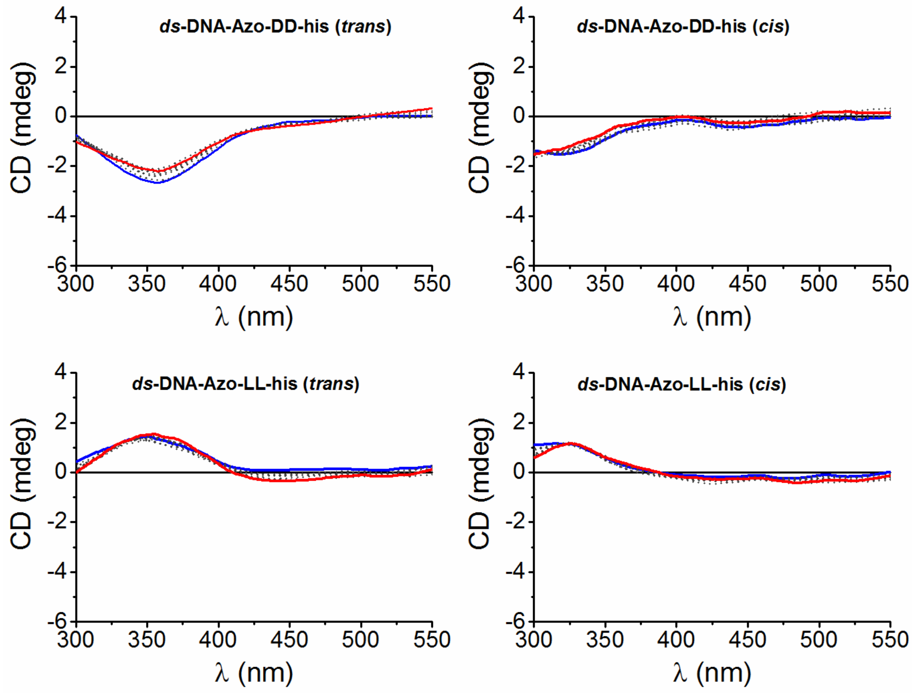


**Figure S31**. ECD spectra resulting from the complexation of **Azo-LL/DD-his** *trans* or *cis*-rich PSS with dsDNA. [**Azo-LL/DD-his** *trans* or *cis*] = 50 μM and [dsDNA] = from 0 to 20 μM. Blue line corresponds to the free ligand and the red line corresponds to the ligand complexed with *ds*-DNA.

# DUPLEX AND QUADRUPLEXES CONFORMATIONAL CHANGES

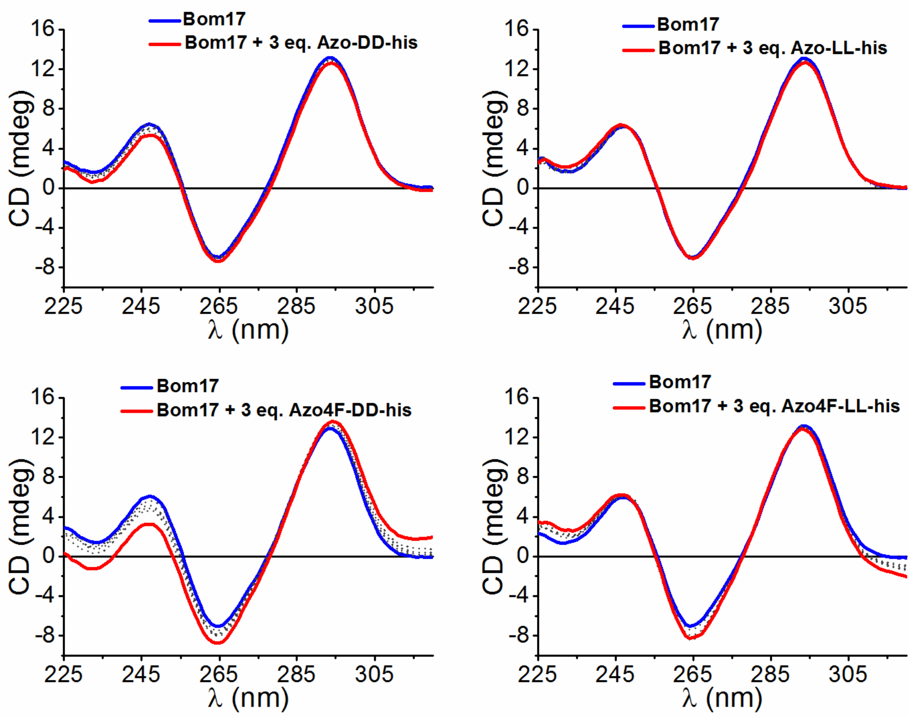


**Figure S32**. ECD spectra of Bom17 upon addition of the azobenzene derivatives in *trans* form. Blue and red lines correspond to the spectra at 0 eq. and 3 eq., respectively. Experimental conditions: $C_{Bom17}$ = 3.0 μM, Tris-HCl 50.0 mM, pH 7.4, KCl 100 mM.

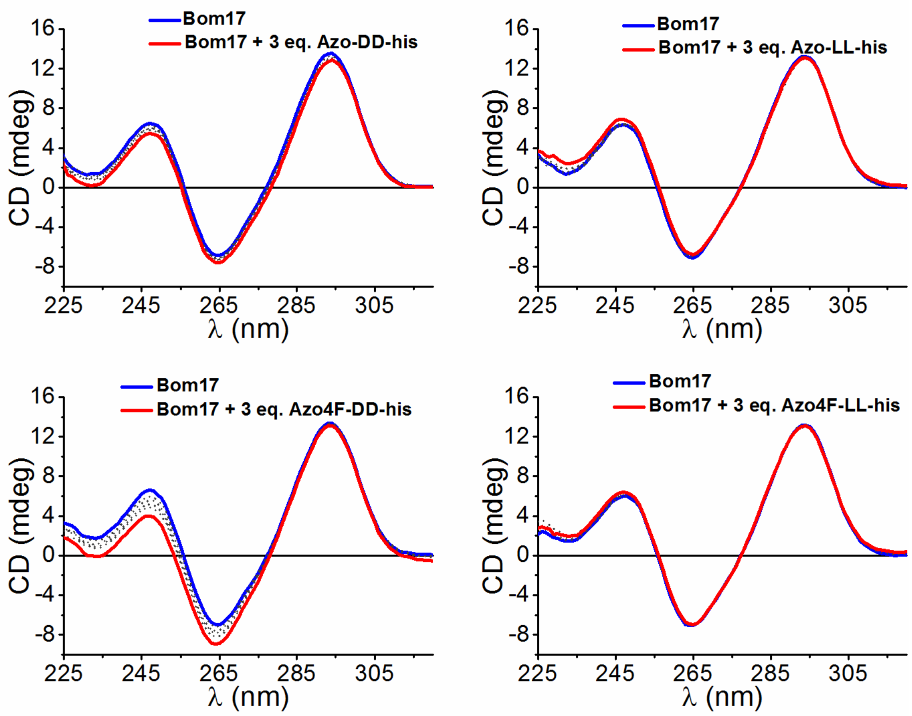


**Figure S33.** ECD spectra of Bom17 upon addition of the azobenzene derivatives as *cis*-rich PSS. Blue and red lines correspond to the spectra at 0 eq. and 3 eq., respectively. Experimental conditions: $C_{Bom17}$ = 3.0 μM, Tris-HCl 50.0 mM, pH 7.4, KCl 100 mM..

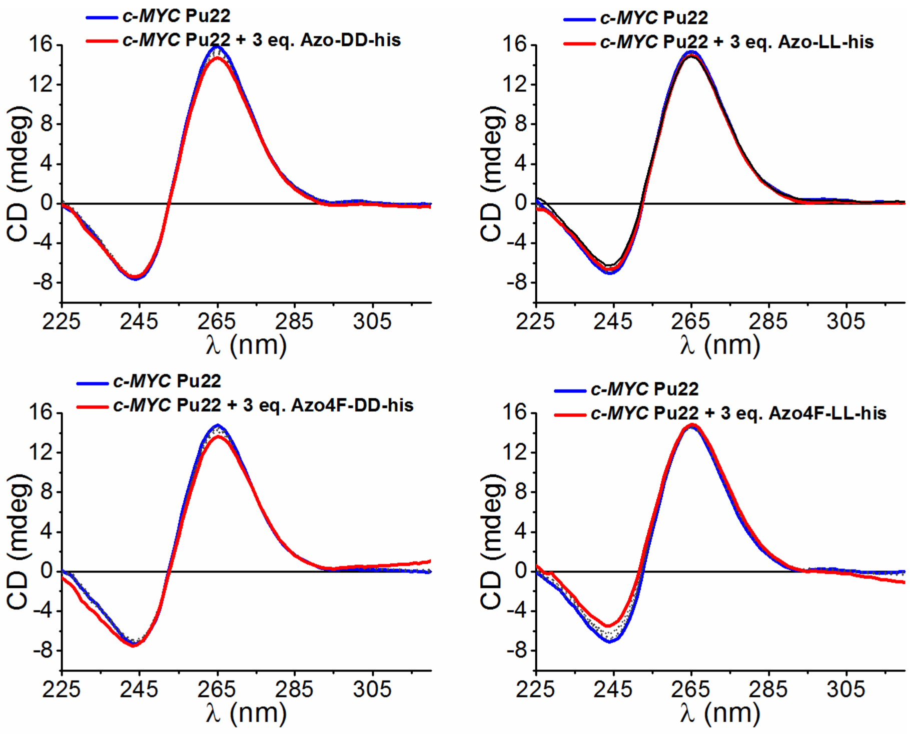


**Figure S34.** ECD spectra of *c-MYC* Pu22 upon addition of the azobenzene derivatives in *trans* form. Blue and red lines correspond to the spectra at 0 eq. and 3 eq., respectively. Experimental conditions: $C_{c\text{-}MYC\ Pu22}$ = 3.0 µM, Tris-HCl 50.0 mM, pH 7.4, KCl 100 mM.

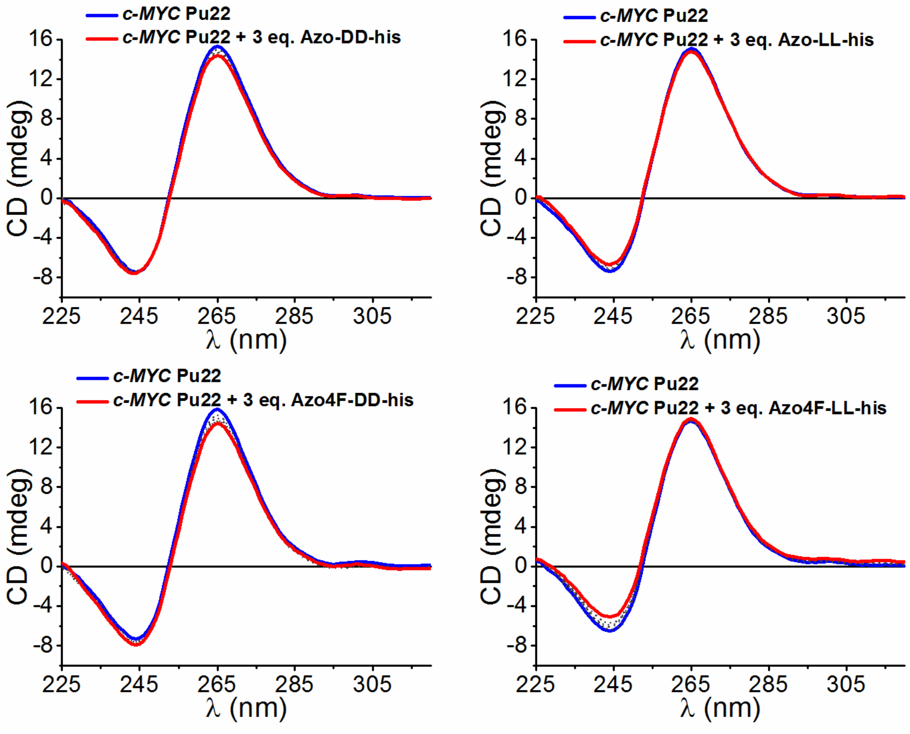


**Figure S35.** ECD spectra of *c-MYC* Pu22 upon addition of the azobenzene derivatives as *cis*-rich PSS. Blue and red lines correspond to the spectra at 0 eq. and 3 eq., respectively. Experimental conditions: $C_{c\text{-}MYC\ Pu22}$ = 3.0 µM, Tris-HCl 50.0 mM, pH 7.4, KCl 100 mM.

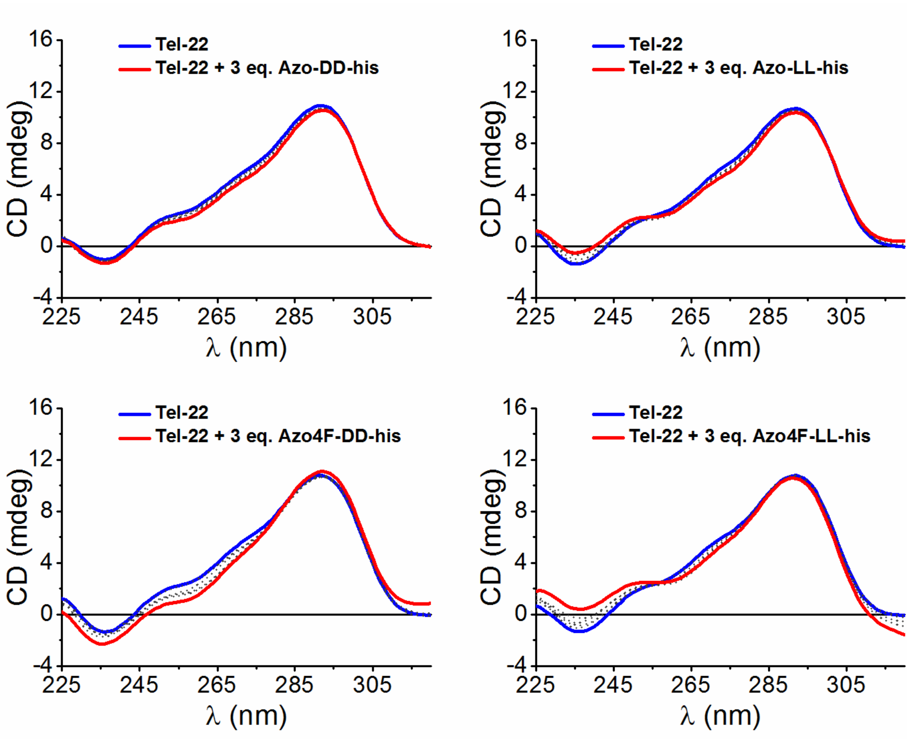


**Figure S36**. ECD spectra of Tel-22 upon addition of the azobenzene derivatives in *trans* form. Blue and red lines correspond to the spectra at 0 eq. and 3 eq., respectively. Experimental conditions: $C_{Tel-22}$ = 3.0 μM, Tris-HCl 50.0 mM, pH 7.4, KCl 100 mM.

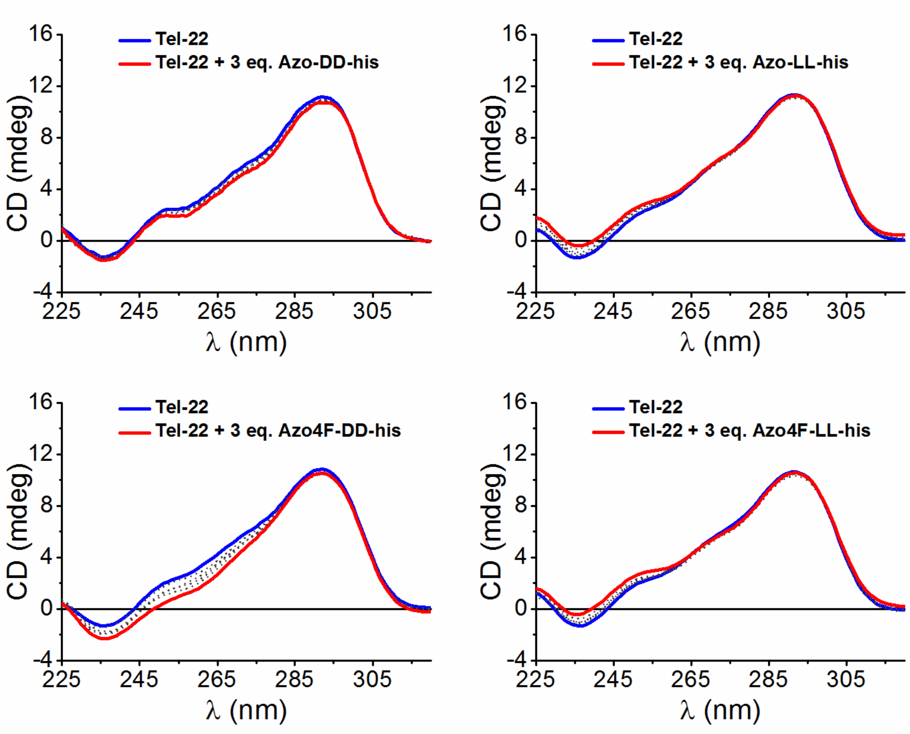


**Figure S37.** ECD spectra of Tel-22 upon addition of the azobenzene derivatives as *cis*-rich PSS. Blue and red lines correspond to the spectra at 0 eq. and 3 eq., respectively. Experimental conditions: $C_{Tel-22}$ = 3.0 μM, Tris-HCl 50.0 mM, pH 7.4, KCl 100 mM.

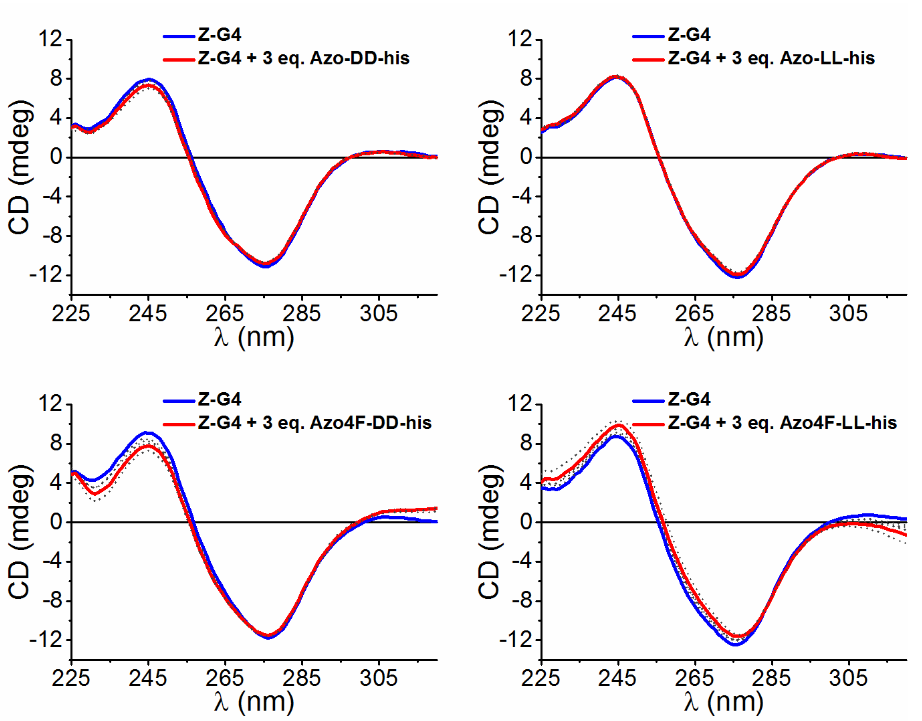


**Figure S38**. ECD spectra of Z-G4 ($K^+$) upon addition of the azobenzene derivatives in *trans* form. Blue and red lines correspond to the spectra at 0 eq. and 3 eq., respectively. Experimental conditions: $C_{Z\text{-}G4}$ = 3.0 µM, Tris-HCl 50.0 mM, pH 7.4, KCl 100 mM.

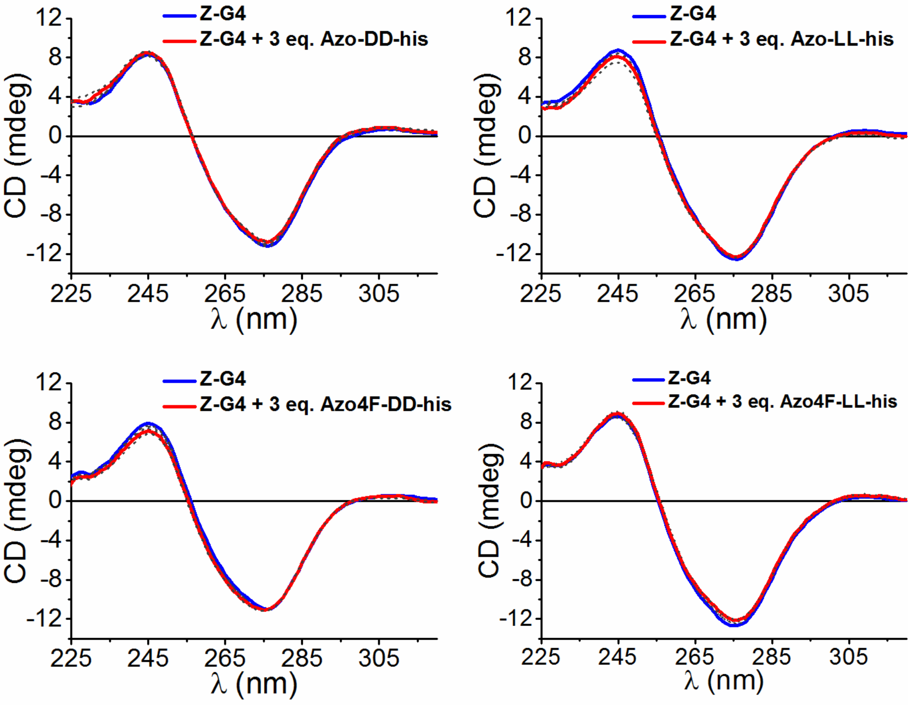


**Figure S39.** ECD spectra of Z-G4 ($K^+$) upon addition of the azobenzene derivatives as *cis*-rich PSS. Blue and red lines correspond to the spectra at 0 eq. and 3 eq., respectively. Experimental conditions: $C_{Z\text{-}G4}$ = 3.0 µM, Tris-HCl 50.0 mM, pH 7.4, KCl 100 mM.

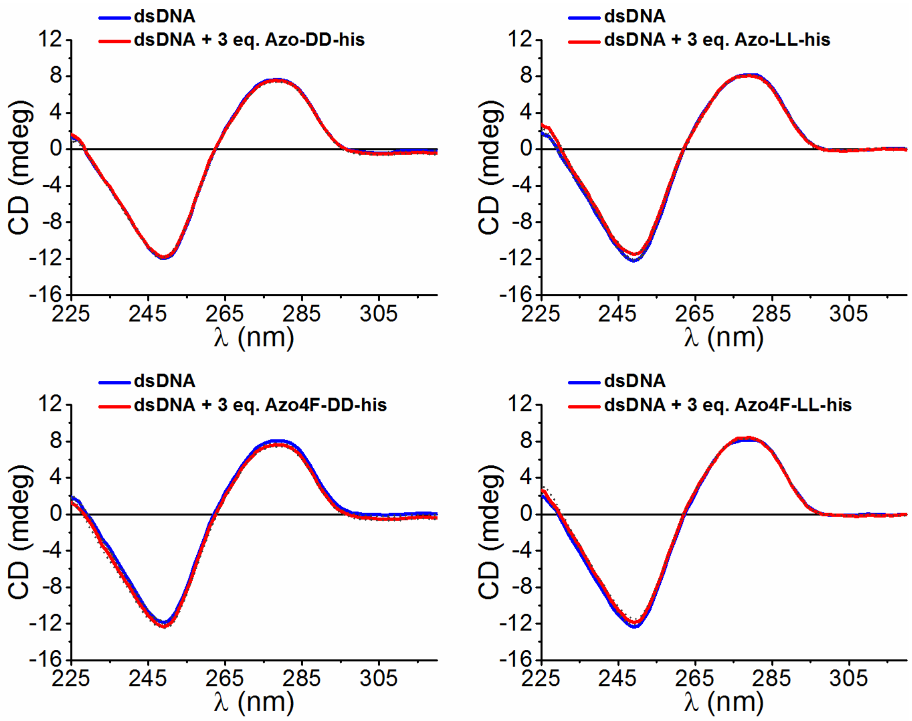


**Figure S40.** ECD spectra of dsDNA($K^+$) upon addition of the azobenzene derivatives in *trans* form. Blue and red lines correspond to the spectra at 0 eq. and 3 eq., respectively. Experimental conditions: $C_{dsDNA}$ = Tris-HCl 50.0 mM, pH 7.4, KCl 100 mM.

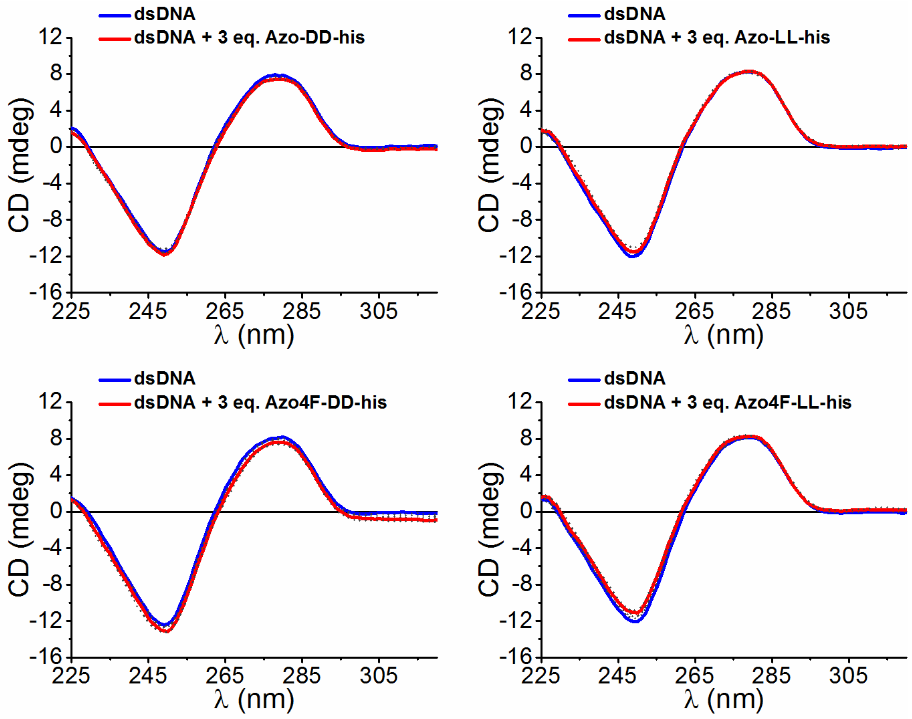


**Figure S41.** ECD spectra of dsDNA($K^+$) upon addition of the azobenzene derivatives as *cis*-rich PSS. Blue and red lines correspond to the spectra at 0 eq. and 3 eq., respectively. Experimental conditions: $C_{dsDNA}$ = Tris-HCl 50.0 mM, pH 7.4, KCl 100 mM.

# THEORETICAL CALCULATIONS

## CONFORMATIONAL SAMPLING

To explore the conformational space of the studied compounds and find the most representative set of the minimum-energy structures having various arrangements, we used a conformational search workflow (iMTD-GC based on metadynamics simulations) implemented in the CREST driver[8] that is a part of the xTB program.[9] The obtained lowest-energy structures were reoptimized at the CPCM/B3LYP-D3BJ/def2-TZVP level of theory[10-12] using the Gaussian 16 package,[13] assuming the water solvent. In the final reoptimized geometries, we found structures featuring the π-stacking interaction of azobenzene and histidine rings (S), formation of NH-π complex (T) or deformation of the histidine ring resulting in a lack of interaction with the azobenzene scaffold (N). In addition, we used protonated amino groups at the side chain of the histidine and deprotonated imidazole ring, which corresponds to the protonation state at pH 7.4. For the selected structures, we computed vertical excitation energies employing the time-dependent density functional theory (TD-DFT), assuming the ωB97xD[14] long-range-corrected functional with the def2-TZVP basis set. The Natural Transition Orbitals (NTOs)[15] analysis was performed in order to determine the character of electronic transitions.

To provide mechanistic insights into the formation of the complex of **Azo-DD-his** *trans* and *c-MYC* Pu22, we assumed a similar computational protocol using the CREST driver, along with the analytical linearized Poisson-Boltzman (ALPB) model, which represents an implicit solvent approach. Our model system was built out for the G-quadruplex 3′-end part (containing only the first layer of guanine bases) and **Azo-DD-his** *trans* that was placed in between the guanine layer and the 3′-end. In addition, we constrained all the atoms of the G-quadruplex 3′-end part and included an implicit solvent model assuming the water solvent. The conformational search workflow allowed us to find the most plausible arrangements of **Azo-DD-his** *trans* in the centre of the G-quadruplex 3′-end part. One of the most stable structures of the complex of **Azo-DD-his** *trans* and the G-quadruplex 3′-end part was reoptimized using the ωB97xD/def2-SVP/PCM method, and all the atoms of the G-quadruplex 3′-end part was constrained. Subsequently, the optimized DFT structure was used to compute vertical excitation energies and simulate UV-Vis and electronic circular dichroism (ECD) spectra (without the G-quadruplex moiety) at the ωB97xD/def2-TZVP/PCM level of theory. The computed ECD spectra line-shapes have been convoluted with Gaussian functions with a HWHM of 0.333 eV.

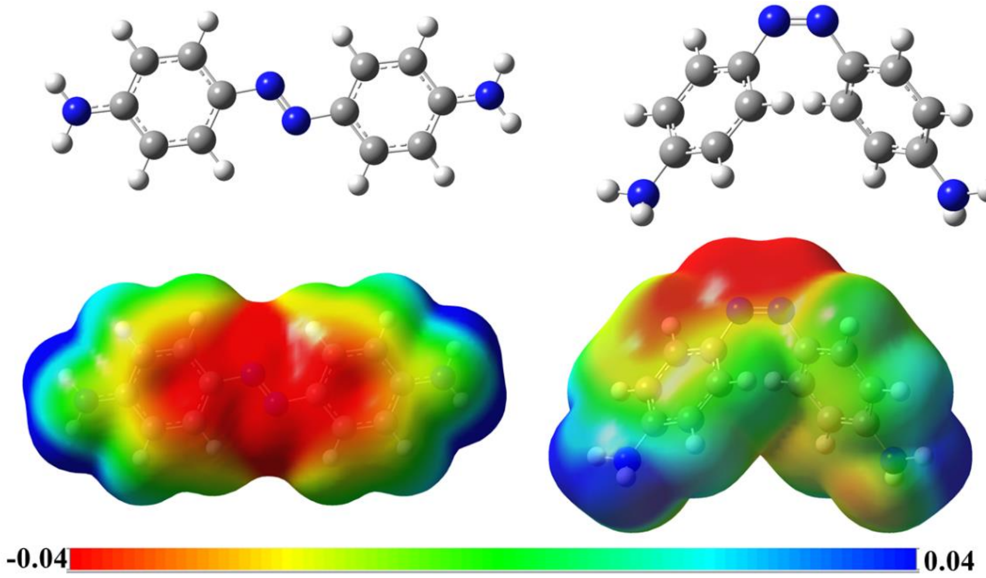


**Figure S42**. *Trans* and *cis* optimized structures (up) and corresponding electrostatic potential (down) for **1**.

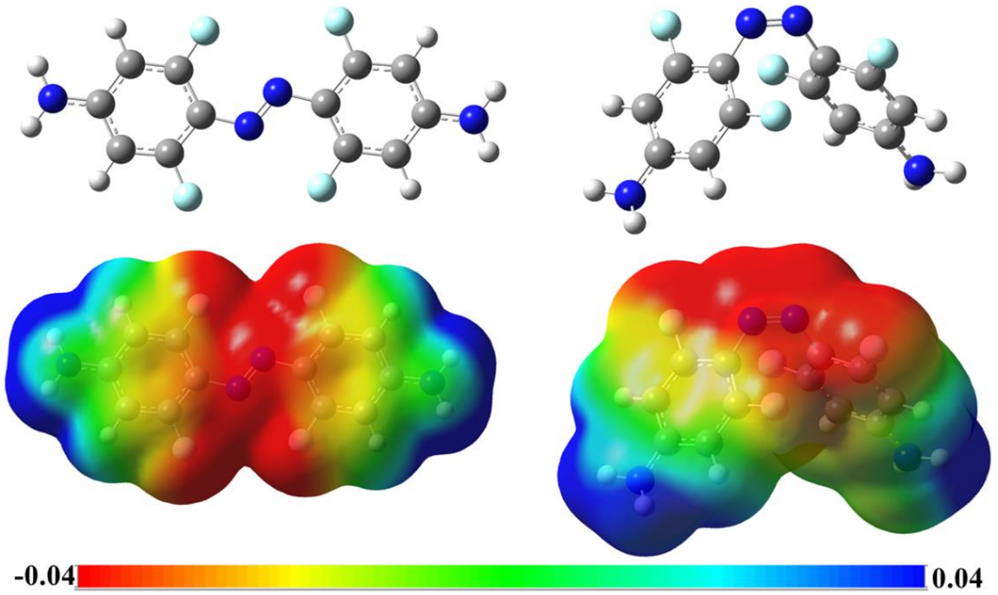


**Figure S43.** *Trans* and *cis* optimized structures (up) and corresponding electrostatic potential (down) for **8**.

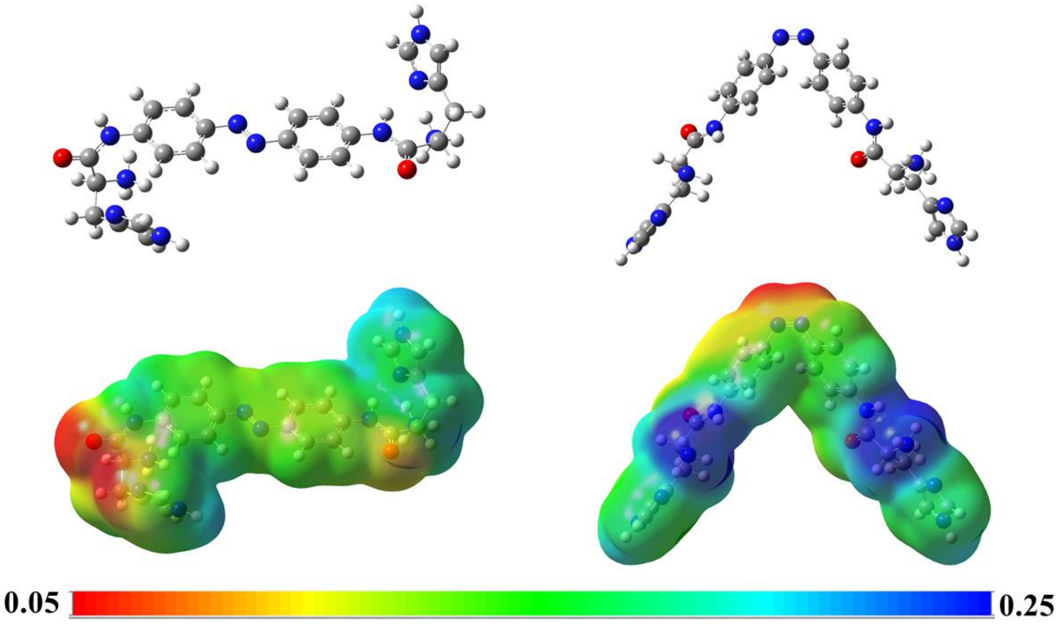


**Figure S44.** *Trans* and *cis* optimized structures (up) and corresponding electrostatic potential (down) for **4** (**Azo-LL-his**).

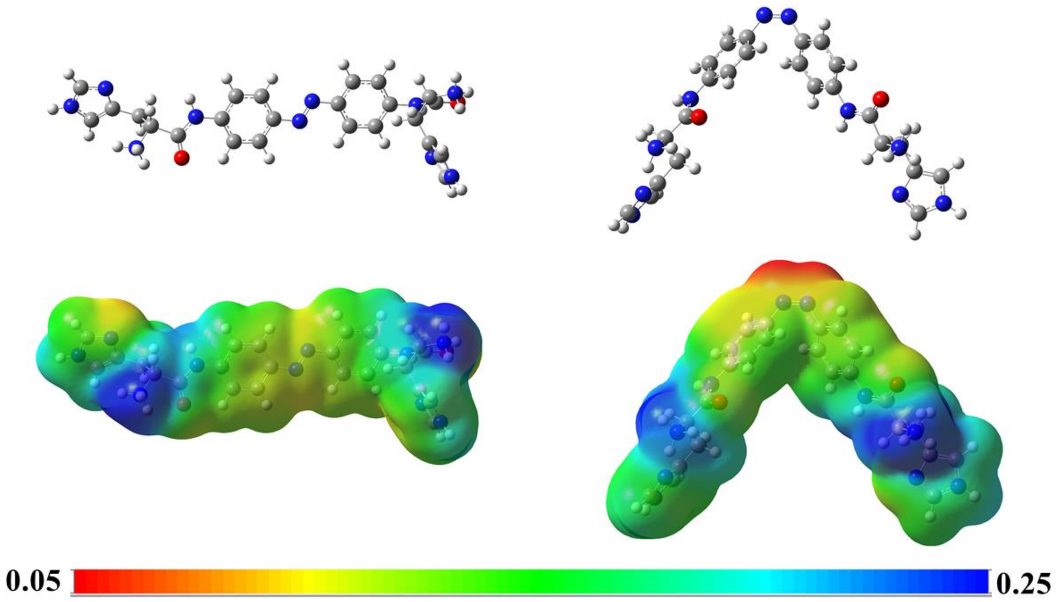


**Figure S45.** *Trans* and *cis* optimized structures (up) and corresponding electrostatic potential (down) for **5** (**Azo-DD-his**).

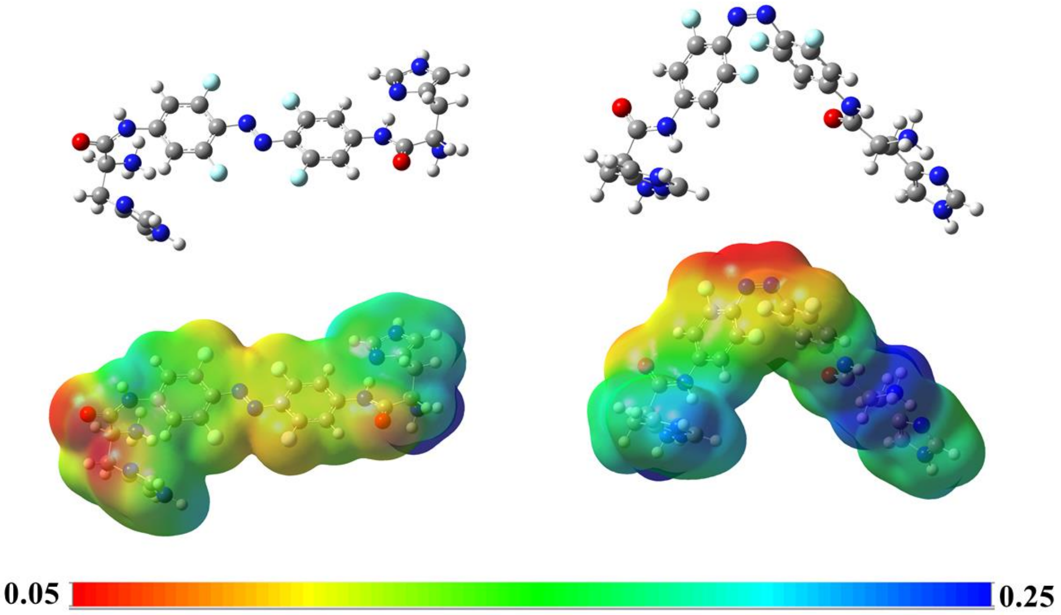


**Figure S46.** *Trans* and *cis* optimized structures (up) and corresponding electrostatic potential (down) for **15** (**Azo4F-LL-his**).

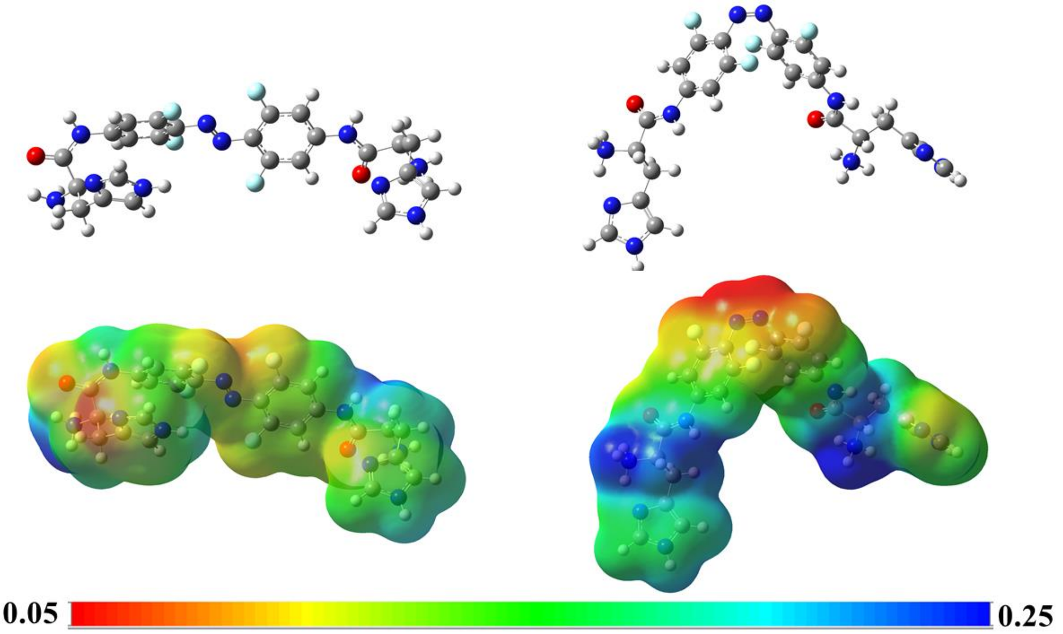


**Figure S47.** *Trans* and *cis* optimized structures (up) and corresponding electrostatic potential (down) for **16** (**Azo4F-DD-his**).

# MOLECULAR DOCKING

Minimized-energy ligand models were flexibly docked on a set of 10 configurations (snapshots) of the 2l7v PDB model using Autodock VINA software,[16] with an embedded energy estimating algorithm (no further improvements were done). This resulted in 200 docking modes for each ligand of **Azo4F-DD-his**, which were further statistically analyzed. The search area for docking fulfilled all accessible binding sites. Distributions of binding energies of ligands interacting with G-quadruplex complex revealed the most probable sites of binding. Structures of each ligand docked to the G4 were optimized further in hybrid method ONIOM[17] as implemented in Gaussian 16, in which **Azo4F-DD-his** in *cis* or *trans* form was optimized at the ωB97xD/6-31++g(d,p)[18] level of theory and the G4 DNA layer assuming PM6 semi-empirical Hamiltonian.[19]

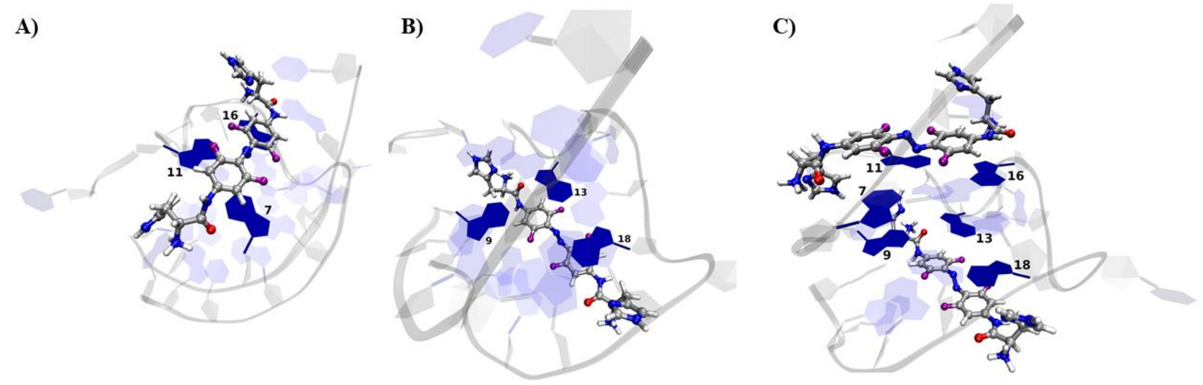


**Figure S48.** Best pose upon docking of compound **Azo4F-DD-his** *trans* and the optimized *c-MYC* Pu22 G4 structure (PDB ID:217v) at (A) 5′-end, (B) 3′-end and (C) both 5′- and 3′-ends. The G4 structure is represented as a wireframe and the ligand as balls and sticks. The guanines highlighted with navy blue indicate the highest affinity towards the ligand.

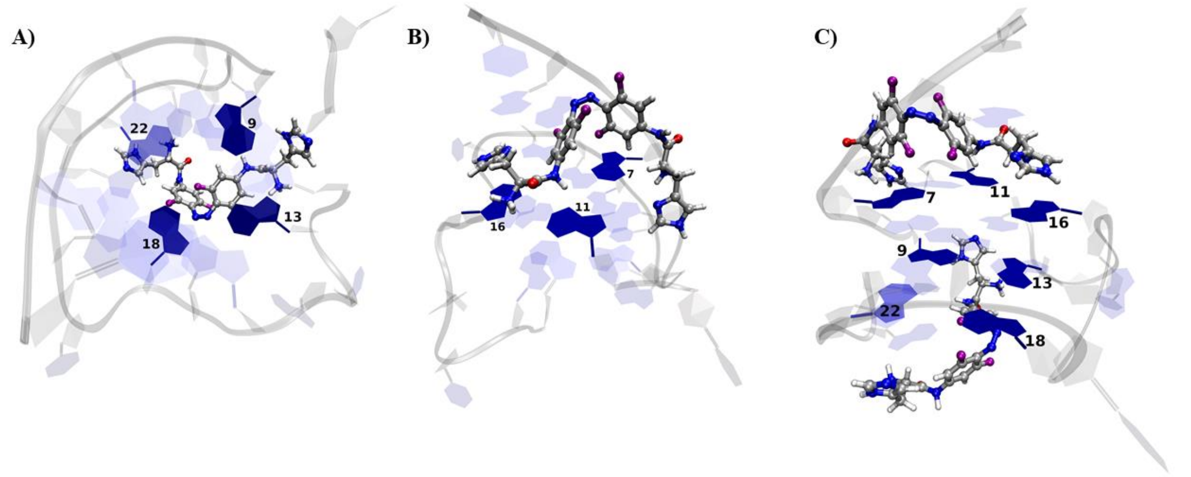


**Figure S49.** Best pose upon docking of compound **Azo4F-DD-his** *cis* and the optimized *c-MYC* Pu22 G4 structure (PDB ID:217v) at (A) 3′-end, (B) 5′-end and (C) both 5′- and 3′-ends. The G4 structure is represented as a wireframe and the ligand as balls and sticks. The guanines highlighted with navy blue indicate the highest affinity towards the ligand.

# REFERENCES


(1) Valeur, E.; Bradley, M. Amide bond formation: beyond the myth of coupling reagents. *Chem. Soc. Rev.* **2009**, *38*, 606-631.

(2) Bleger, D.; Schwarz, J.; Brouwer, A. M.; Hecht, S. o-Fluoroazobenzenes as readily synthesized photoswitches offering nearly quantitative two-way isomerization with visible light. *J. Am. Chem. Soc.* **2012**, *134*, 20597-600.

(3) Deiana, M.; Pokladek, Z.; Dudek, M.; Mucha, S. G.; Mazur, L. M.; Pawlik, K.; Mlynarz, P.; Samoc, M.; Matczyszyna, K. Remote-control of the enantiomeric supramolecular recognition mediated by chiral azobenzenes bound to human serum albumin. *Phys.Chem.Chem.Phys.* **2017**, *19*, 21272-21275

(4) Zhang, Z.; Dai, J.; Veliath, E.; Jones, R. A.; Yang, D. Structure of a two-G-tetrad intramolecular G-quadruplex formed by a variant human telomeric sequence in $K^+$ solution: insights into the interconversion of human telomeric G-quadruplex structures. *Nucleic Acids Res.* **2009**, *38*, 1009-1021.

(5) De Rache, A.; Mergny, J.-L. Assessment of selectivity of G-quadruplex ligands via an optimised FRET melting assay. *Biochimie* **2015**, *115*, 194-202.

(6) Largy, E.; Mergny, J.-L. Shape matters: size-exclusion HPLC for the study of nucleic acid structural polymorphism. *Nucleic Acids Res.* **2014**, *42*, e149.

(7) Chung, W. J.; Heddi, B.; Schmitt, E.; Lim, K. W.; Mechulam, Y.; Phan, A. T. Structure of a left-handed DNA G-quadruplex. *Proc. Natl. Acad. Sci. U.S.A.* **2015**, *112*, 2729-2733.

(8) Pracht, P.; Bohle, F.; Grimme, S. Automated exploration of the low-energy chemical space with fast quantum chemical methods. *Phys.Chem.Chem.Phys.* **2020**, *22*, 7169-7192.

(9) Bannwarth, C.; Caldeweyher, E.; Ehlert, S.; Hansen, A.; Pracht, P.; Seibert, J.; Spicher, S.; Grimme, S. Extended tight-binding quantum chemistry methods. *WIREs Comput. Mol. Sci.* **2021**, *11*, e1493.

(10) Becke, A. D. Density-functional thermochemistry. III. The role of exact exchange. *J. Chem. Phys.* **1993**, *98*, 5648-5652.

(11) Grimme, S.; Ehrlich, S.; Goerigk, L. Effect of the damping function in dispersion corrected density functional theory. *J. Comp. Chem.* **2011**, *32*, 1456-1465.

(12) Weigend, F.; Ahlrichs, R. Balanced basis sets of split valence, triple zeta valence and quadruple zeta valence quality for H to Rn: Design and assessment of accuracy. *Phys.Chem.Chem.Phys.* **2005**, *7*, 3297-3305.

(13) Frisch, M. J.; Trucks, G. W.; Schlegel, H. B.; Scuseria, G. E.; Robb, M. A.; Cheeseman, J. R.; Scalmani, G.; Barone, V.; Petersson, G. A.; Nakatsuji, H.; Li, X.; Caricato, M.; Marenich, A. V.; Bloino, J.; Janesko, B. G.; Gomperts, R.; Mennucci, B.; Hratchian, H. P.; Ortiz, J. V.; Izmaylov, A. F.; Sonnenberg, J. L.; Williams; Ding, F.; Lipparini, F.; Egidi, F.; Goings, J.; Peng, B.; Petrone, A.; Henderson, T.; Ranasinghe, D.; Zakrzewski, V. G.; Gao, J.; Rega, N.; Zheng, G.; Liang, W.; Hada, M.; Ehara, M.; Toyota, K.; Fukuda, R.; Hasegawa, J.; Ishida, M.; Nakajima, T.; Honda, Y.; Kitao, O.; Nakai, H.; Vreven, T.; Throssell, K.; Montgomery Jr., J. A.; Peralta, J. E.; Ogliaro, F.; Bearpark, M. J.; Heyd, J. J.; Brothers, E. N.; Kudin, K. N.; Staroverov, V. N.; Keith, T. A.; Kobayashi, R.; Normand, J.; Raghavachari, K.; Rendell, A. P.; Burant, J. C.; Iyengar, S. S.; Tomasi, J.; Cossi, M.; Millam, J. M.; Klene, M.; Adamo, C.; Cammi, R.; Ochterski, J. W.; Martin, R. L.; Morokuma, K.; Farkas, O.; Foresman, J. B.; Fox, D. J.: Gaussian 16 Rev. C.01. Wallingford, CT, 2016.

(14) Chai, J.-D.; Head-Gordon, M. Long-range corrected hybrid density functionals with damped atom–atom dispersion corrections. *Phys.Chem.Chem.Phys.***2008**, *10*, 6615-6620.

(15) Martin, R. L. Natural transition orbitals. *J. Chem. Phys.* **2003**, *118*, 4775-4777.

(16) Trott, O.; Olson, A. J. AutoDock Vina: Improving the speed and accuracy of docking with a new scoring function, efficient optimization, and multithreading. *J. Comp. Chem.* **2010**, *31*, 455-461.
(17) Dapprich, S.; Komáromi, I.; Byun, K. S.; Morokuma, K.; Frisch, M. J. A new ONIOM implementation in Gaussian98. Part I. The calculation of energies, gradients, vibrational frequencies and electric field derivatives. *J. Mol. Struct.* **1999**, *461-462*, 1-21.
(18) Petersson, G. A.; Al-Laham, M. A. A complete basis set model chemistry. II. Open-shell systems and the total energies of the first-row atoms. *J. Chem. Phys.* **1991**, *94*, 6081-6090.
(19) Stewart, J. J. P. Optimization of parameters for semiempirical methods V: Modification of NDDO approximations and application to 70 elements. *J. Mol. Model.* **2007**, *13*, 1173-1213.
